# Radiomics-enhanced modelling approach for predicting the need for ECMO in ARDS patients: a retrospective cohort study

**DOI:** 10.1038/s41598-025-21287-w

**Published:** 2025-09-30

**Authors:** Martin Mirus, Eric Leitert, Rebecca Bockholt, Lars Heubner, Steffen Löck, Marie Brei, Jonas Biehler, Jens-Peter Kühn, Thea Koch, Wolfgang Wall, Peter Markus Spieth

**Affiliations:** 1 Department of Anaesthesiology and Intensive Care Medicine, Faculty of Medicine and University Hospital Carl Gustav Carus, TUD Dresden University of Technology, Fetscherstrasse 74, 01307 Dresden, Germany; 2 OncoRay – National Center for Radiation Research in Oncology, Faculty of Medicine and University Hospital Carl Gustav Carus, TUD Dresden University of Technology, Fetscherstrasse 74, 01307 Dresden, Germany; 3 Institute for Computational Mechanics, Technical University Munich, Boltzmannstraße 15, 85748 Munich, Germany; 4 Ebenbuild GmbH, Holzstraße 28, 80469 Munich, Germany; 5 Department of Diagnostic and Interventional Radiology, Faculty of Medicine and University Hospital Carl Gustav Carus, TUD Dresden University of Technology, Fetscherstrasse 74, 01307 Dresden, Germany

**Keywords:** ECMO, Extracorporeal membrane oxygenation, Prediction, CNN, Convolutional neural network, Lung segmentation, CT, Computed tomography, Critical care, Decision making, Radiomic, Medical imaging, Respiratory distress syndrome

## Abstract

Decisions regarding veno-venous extracorporeal membrane oxygenation (vv-ECMO) in patients with acute respiratory distress syndrome (ARDS) are often based solely on clinical and physiological parameters, which may insufficiently reflect severity and heterogeneity of lung injury. This study aimed to develop a predictive model integrating machine learning-derived quantitative features from admission chest computed tomography (CT) with selected clinical variables to support early individualized decision-making regarding vv-ECMO therapy. In this retrospective single-center cohort study, 375 consecutive patients with COVID-19-associated ARDS admitted to the ICU between March 2020 and April 2022 were included. Lung segmentation from initial CTs was performed using a convolutional neural network (CNN) to generate high-resolution, anatomically accurate masks of the lungs. Subsequently, 592 radiomic features, quantifying lung aeration, density and morphology, were extracted. Four clinical parameters – age, mean airway pressure, lactate, and C-reactive protein, were selected on the basis of clinical relevance. Three logistic regression models were developed: (1) Imaging Model, (2) Clinical Model, and (3) Combined Model integrating different features. Predictive performance was assessed via the area under the receiver operating characteristic curve (AUROC), accuracy, sensitivity, and specificity. A total of 375 patients were included: 172 in the training and 203 in the validation cohort. In the training cohort, the AUROCs were 0.743 (Imaging), 0.828 (Clinical), and 0.842 (Combined). In the validation cohort, the Combined Model achieved the highest AUROC (0.705), outperforming the Clinical (0.674) and Imaging (0.639) Models. Overall accuracy in the validation cohort was 64.0% (Combined), 66.5% (Clinical), and 59.1% (Imaging). The Combined Model showed 68.1% sensitivity and 58.9% specificity. Kaplan-Meier analysis confirmed a significantly greater cumulative incidence of ECMO therapy in patients predicted as high risk (*p* < 0.001), underscoring its potential to support individualized, timely ECMO decisions in ARDS by providing clinicians with objective data-driven risk estimates. Quantitative CT features based on machine learning-derived lung segmentation allow early individualized prediction of the need for vv-ECMO in ARDS. While clinical data remain essential, radiomic markers enhance prognostic accuracy. The Combined Model demonstrates considerable potential to support timely and evidence-based ECMO initiation, facilitating individualized critical care in both specialized and general ICU environments.

**Trial registration**: The study is registered with the German Clinical Trials Register under the number DRKS00027856. Registered 18.01.2022, retrospectively registered due to retrospective design of the study.

**Supplementary Information:**

The online version contains supplementary material available at 10.1038/s41598-025-21287-w.

## Introduction

The decision to initiate veno-venous extracorporeal membrane oxygenation (vv-ECMO), a life-saving intervention for patients with severe acute respiratory distress syndrome (ARDS)^1–3^, remains complex and time-sensitive, especially in hospitals with limited ECMO experience. Several scoring systems, such as the RESP score and the PRESERVE score, have been proposed to guide this decision^4–6^. However, these models were primarily designed to predict outcomes after ECMO initiation and rely largely on clinical and physiological variables assessed at or near the time of cannulation^7–9^. Consequently, they offer only limited support for the early identification of patients who may later become ECMO candidates.

In contrast, early identification of such patients, ideally at ICU admission, may enable timely vv-ECMO initiation, optimize resource use, support proactive, individualized treatment planning, and facilitate timely referrals to specialized ECMO centers, avoiding both unnecessary transfers and delayed therapy. Machine learning approaches based on clinical data have shown promise in predicting ARDS phenotypes and trajectories^10,11^, but their performance may be limited by the lack of morphological and pathophysiological information available in imaging data. Quantitative chest CT analysis represents an underutilized yet highly informative resource. CT scans are routinely performed in ARDS patients early in their clinical course, but standard radiology reports offer only qualitative, often subjective, information. In contrast, quantitative imaging, especially when automated through convolutional neural networks (CNNs)^12,13^, can extract reproducible features that capture the distribution of lung aeration, density, and geometry^14–16^. These imaging biomarkers may provide complementary information to clinical parameters, potentially improving predictive performance. Previous studies have shown that CT-based metrics such as lung weight, aeration, and heterogeneity are associated with ARDS severity and ventilation response^15,16^, but these metrics have not yet been applied to predict future ECMO requirement. Moreover, while quantitative imaging has been proposed for determining the recruitment potential or PEEP setting, its value in supporting early triage decisions for ECMO has not been systematically evaluated.

## Methods

This publication adheres to the Transparent Reporting of a Multivariable Prediction Model for Individual Prognosis or Diagnosis (TRIPOD + AI statement)^17^.

### Study aim

This study aimed to develop and validate a model for predicting vv-ECMO requirements in patients with ARDS secondary to COVID-19 at the time of intensive care unit (ICU) admission by integrating machine learning-derived quantitative CT features with key clinical parameters. We hypothesized that the incorporation of radiomic imaging biomarkers would increase prognostic precision and support individualized, data-driven decision-making regarding timely initiation of ECMO therapy.

## Study design

A retrospective single-center study was conducted including two ARDS cohorts admitted to the ICU: a training cohort for model development and a validation cohort for model validation. To predict the need for vv-ECMO, three logistic regression models were developed:

1. Imaging Model: Trained on CT-derived imaging features.
2. Clinical Model: Trained on clinical features.
3. Combined Model: This model integrates both imaging and clinical features.

**Fig. 1:**
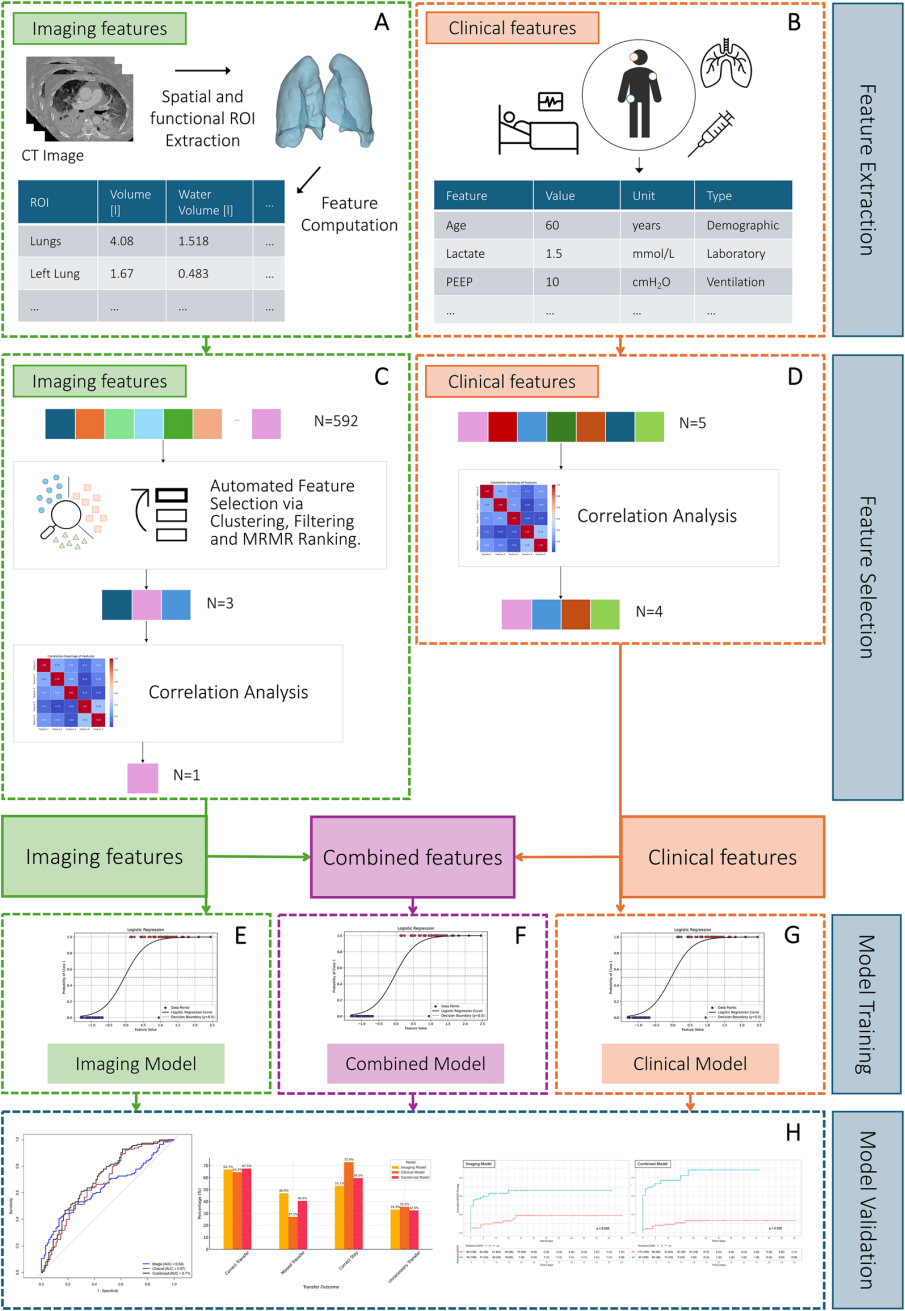
Study design: Model development for predicting veno-venous ECMO in ARDS patients. This figure depicts the process of developing and validating three machine learning models for predicting the need for vv-ECMO in ARDS patients. In the feature extraction phase, 592 imaging features were extracted through automated CT segmentation and quantitative image analysis (**A**), and five clinical features were obtained from electronic health records (**B**). During the feature selection phase, relevant features were selected. For imaging, a multi-step feature selection process including clustering, cross-validated Minimum Redundancy Maximum Relevance (MRMR) ranking and correlation analysis were performed (**C**). Clinical parameters were assessed for selection on the basis of correlation analysis (**D**). The selected features formed the basis for the training of the Imaging Model (**E**) and the Clinical Model (**G**). These feature sets were then combined to train the Combined Model (**F**). Finally, all the models were validated in the validation cohort (**H**).

Model development followed a structured, multistep pipeline involving feature extraction, selection, and training, as illustrated in Fig. 1, where the overall workflow is depicted. First, imaging features were extracted from CT scans, whereas clinical features were obtained from patients’ medical records. Next, feature selection was performed to identify key imaging and clinical features for the development of the three logistic regression models. Logistic regression was chosen as the underlying model because of its interpretability and effectiveness in binary classification tasks. The models were trained and evaluated via standard classification metrics, and their ability to predict the future need for vv-ECMO support was assessed. Informed consent was waived in accordance with German legal regulations, and this approach was approved by the competent ethics committee at the Technical University of Dresden (BO-EK-374072021). The study is registered in the German Clinical Trials Register (DRKS00027856).

### Data collection and inclusion

All patients admitted to the Intensive Care Unit (ICU) of the study center between March 19, 2020, and up to including March 10, 2021, were screened for inclusion in the training cohort, whereas those admitted from March 11, 2021, and March 29, 2022 were screened for inclusion in the validation cohort. The inclusion criteria were admission to the ICU, ARDS according to the Berlin definition^18^, and confirmed COVID-19 infection. There were no exclusion criteria. Clinical data were recorded within 30 min after admission. The ventilator settings were recorded on admission if the patient was already intubated or within a range of 30 min after intubation. For the laboratory parameters included in this study, the value at admission was used. If multiple values were available at admission, the mean of these values was calculated. For lactate, the value from the first blood gas analysis at admission was used. The CT scan was performed in close temporal proximity to the ICU admission at the study center, either on site or at the referring hospital.

### Imaging model: feature extraction

**Fig. 2:**
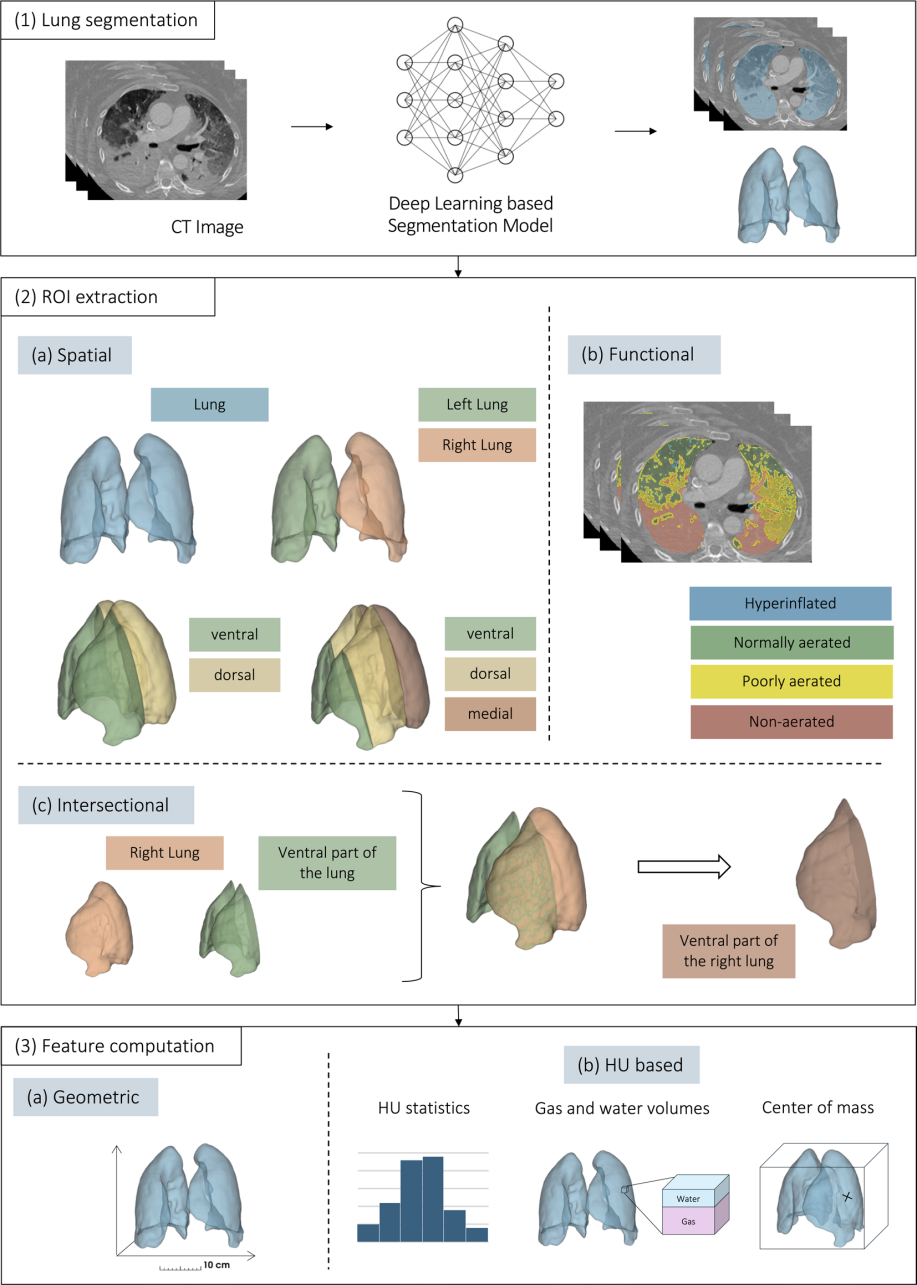
Three-phase process of quantitative image analysis for feature extraction. The figure illustrates the three phases of quantitative image analysis for feature extraction from CT images. (1). Lung segmentation: The lungs are first segmented from the CT scan to define the overall analysis region. (2). ROI extraction: Within the segmented lungs, ROIs are extracted in three categories: spatial (**a**) (e.g., anatomical compartments), functional (**b**) (e.g., aeration-based regions), and intersectional (c) (overlaps between spatial and functional ROIs). (3). Feature computation: Quantitative features are extracted from each ROI, including geometrical features (**a**) to describe the shape and structure of each ROI and HU-based characteristics (**b**) to quantify the tissue density in each ROI. CT: computed tomography; ROI: region of interest; HU: Hounsfield unit.

The quantitative CT analysis in this study enabled the extraction of 592 objective and reproducible features to quantify the imaged lung in a time-efficient manner^14^. This analysis is illustrated in Fig. 2 and can be broadly divided into three phases: (1) Lung segmentation; (2) Extraction of regions of interest (ROIs) within the lung; (3) Computation of quantitative features within these regions.

#### Lung segmentation

The initial step was to delineate the lung boundaries in the CT scan. For this purpose, an in-house segmentation model based on published CNN-based network architectures^12,13^ was used. Prior to applying the segmentation model, the CT scans were downscaled to a resolution of 256 × 256 × 128 voxels to ensure consistent input dimensions. Furthermore, voxel intensities (Hounsfield units, HUs) were scaled to zero mean and unit variance to account for interscan intensity differences and promote comparability. To restore the spatial resolution of the CT images for the quantitative analysis, the resulting lung masks were subsequently upsampled to the original resolution of the CT images and underwent postprocessing, including morphological closing to fill small holes in the segmentation mask and connected component analysis to remove isolated artifacts. To ensure consistent, high-quality segmentation, all the lung masks were manually reviewed and corrected in a blinded fashion by clinicians experienced in thoracic imaging and trained in the interpretation of the lung CT scans.

#### ROI extraction

**Fig. 3:**
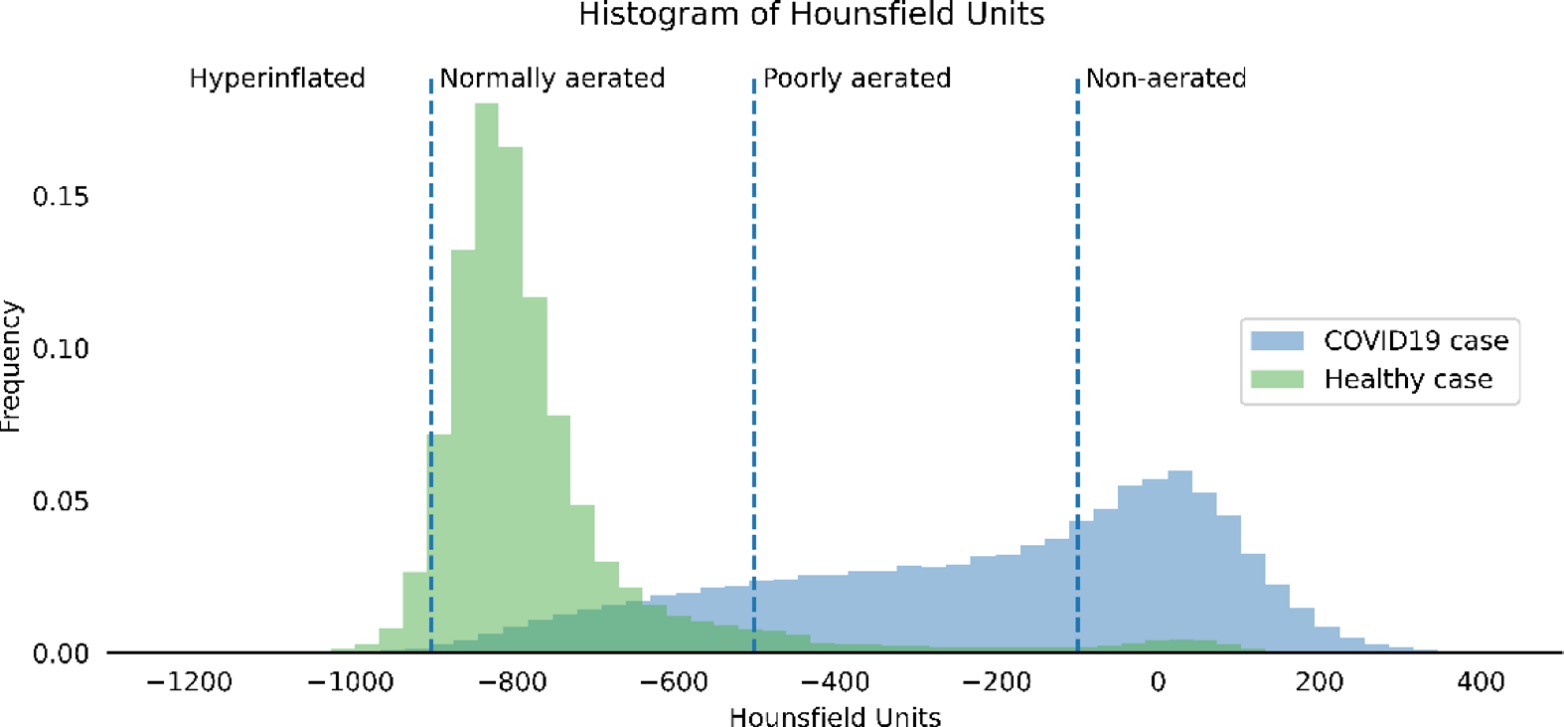
Hounsfield units (HUs) within different lung areas. Exemplary representation of the frequency of different HUs in the lungs of a COVID-19 patient (blue) from this study. For comparison, a healthy control (green) is included. The dashed vertical lines indicate the HU intervals used to subdivide the lungs into regions of different aeration conditions: hyperinflated, normally aerated, poorly aerated, and nonaerated^15^. The frequency of HUs in the different areas differs between the two cases shown. While for the healthy controls (green), most HUs fell within the range of normal aeration, the COVID-19 patients exhibited a broader distribution extending into regions associated with poor and no aeration. HU: Hounsfield units.

Specific ROIs within the segmented lung mask were extracted. These include spatial ROIs, which divide the lung into anatomically distinct compartments; functional ROIs, which categorize lung areas on the basis of aeration characteristics; and intersectional ROIs, which capture the overlap between different spatial and functional regions. For the spatial ROIs, the segmented lung mask, comprising both the left and right lungs as a single compartment, was defined as the primary ROI. To further differentiate anatomical structures, separate masks for the right and left lungs were extracted. Furthermore, the lungs were subdivided along the ventral-dorsal axis using two alternative strategies: a two- (ventral and dorsal ) and three-compartment division (ventral, medial, and dorsal ). For the two-compartment division the lung was sliced along the coronal plane through its centroid, separating it into a ventral and dorsal region. For the three-compartment division, the lung was first enclosed within a 3D bounding box. This box was then evenly partitioned along the ventral‒dorsal axis, resulting in three spatially distinct regions: the ventral, medial, and dorsal compartments. To extract the functional ROIs, the lung mask was partitioned into four compartments, namely, a hyperinflated, normally aerated, poorly aerated, and nonaerated area, on the basis of the HU ranges, as defined by Gattinoni et al.^15^ and visualized in Fig. 3. To further analyze spatial relationships, the intersections between different spatial and functional ROIs, such as the overlap of the left lung with, e.g., the ventral compartment or nonaerated area of the lung, were computed. In total, 42 spatial, functional and intersectional ROIs within the lung were extracted (Table S1).

#### Feature computation

This involves estimating quantitative characteristics within the extracted ROIs. For each ROI, 14 quantitative imaging features, which are listed in Table 1, were calculated to capture the intensity and geometric characteristics of the ROIs.

More specifically, to quantify the geometric properties and describe the shape and structure of each ROI, the overall volume of each ROI (*V*_*ROI*_) was computed by

1 $$\:{V}_{\text{ROI}}{[mm}^{3}]={V}_{voxel}{[mm}^{3}]*N{V}_{ROI}$$

where $\:N{V}_{\:ROI}\:$ denotes the number of voxels within the ROI mask and where $\:{V}_{voxel}$ represents the volume of a single voxel. In addition, the spatial dimensions of each ROI were calculated along the transverse, sagittal and longitudinal axes. To quantitatively assess the lung tissue density and composition of each ROI, ten HU-based features were computed within each ROI. These features included, among others, the mean, standard deviation, kurtosis, and skewness of the voxel HU intensity values, capturing key statistical properties of their distribution. Furthermore, the water and gas volume as well as the weight of the lung parenchyma within each ROI were estimated via the relationship between CT attenuation and the physical density encoded in the HU values^15,19^. More specifically, for this study, the simplifying assumption was adopted that each voxel represents a mixture of gas and water where the water component consists of lung tissue, blood, extravascular fluid, and cellular debris, whereas the gas component corresponds to the aerated portion of the voxel. Following this assumption and the approach of Protti et al.^16^, the gas and water volumes of each ROI were computed. Prior to this, the volumetric gas fraction of each voxel was estimated as follows:

2 $$\:{V}_{\text{gas,\:voxel}}\:\left[{mm}^{3}\right]=\frac{{HU}_{voxel}}{{HU}_{air\:}}*{V}_{voxel}{[mm}^{3}]$$

where $\:{HU}_{air\:}$ is defined as -1000 HU.

The gas volume within an ROI was obtained by summing the contributions of individual voxels within that ROI:

3 $$\:{V}_{\text{gas,\:ROI}}\:\left[{mm}^{3}\right]={\sum\:}_{i=1}^{{NV}_{ROI}}{V}_{gas,\:\:voxel,\:i}$$

where $\:i$ explicitly refers to individual voxels within the ROI.

Following the assumption that each voxel is a mixture of gas and water, the water volume was estimated by subtracting the gas volume from the total ROI volume:

4 $$\:{V}_{water\text{,\:ROI}}\:\left[{mm}^{3}\right]={V}_{ROI}\left[{mm}^{3}\right]-{V}_{\text{gas,\:ROI}}\:\left[{mm}^{3}\right]$$

The weights of the lung tissue for the ROIs were then estimated as follows:

5 $$\:{W}_{\text{\:tissue,\:ROI}}\:\:\left[g\right]={V}_{water\text{,\:ROI}}\:\left[{mm}^{3}\right]*9.933{e}^{-4}\left[\frac{g}{{mm}^{3}}\right]$$

where $\:9.933{e}^{-4}$ is the density of water in $\:\frac{g}{{mm}^{3}}$ at $\:37\:^\circ\:C.$

Furthermore, the center of gravity (COG) was determined for each ROI relative to the lung boundary. Finally, the absolute volume of the aeration compartments in the lung, i.e., hyperinflated, normally aerated, poorly aerated and nonaerated, was converted into relative quantities, expressed as a fraction of the overall volume of the lung. This process introduces four additional features, complementing the existing 14 features per ROI. As a result, a total of 592 quantitative features were extracted, providing a comprehensive characterization of lung structure and function across all the ROIs.

**Table 1:** Overview of quantitative imaging features and their descriptions.

Features	Name	Description
HU based characteristics	Mean	Mean value of the distribution of HU values in a ROI.
	Standard deviation	Standard deviation of the distribution of HU values in a ROI.
	Kurtosis	Kurtosis of the distribution of HU values in a ROI.
	Skewness	Skewness of the distribution of HU values in a ROI.
	Gas volume	Gas volume of a ROI (in $\:{mm}^{3}$) according to formula (3).
	Water volume	Water volume of a ROI (in $\:{mm}^{3}$) according to formula (4).
	Tissue weight	Tissue weight of a ROI (in g) according to formula (5).
	Relative position of the center of mass; transversal	Position of the HU-weighted center of mass of a ROI along the transversal axis from right to left, relative to the boundary of the lung mask.
	Relative position of the center of mass; sagittal	Position of the HU-weighted center of mass of a ROI along the sagittal axis from ventral to dorsal, relative to the boundary of the lung mask.
	Relative position of the center of mass; longitudinal	Position of the HU-weighted center of mass of a ROI along the longitudinal axis from caudal to cranial, relative to the boundary of the lung mask.
Geometric characteristics	Volume	Volume of a ROI (in $\:{mm}^{3}$) according to formula (1).
	Transversal dimension	Spatial dimensions of a ROI (in $\:mm)$ along the transversal axis.
	Sagittal dimension	Spatial dimensions of a ROI (in $\:mm)$ along the sagittal axis.
	Longitudinal dimension	Spatial dimensions of a ROI (in $\:mm)$ along the longitudinal axis.

Quantitative imaging features. The table shows both HU-based and geometric characteristics, both of which are extracted from CT images via quantitative image analysis. Formulas (1, 3–5) referred to in the table are found within the methods section. HU: Hounsfield unit; ROI: region of interest.

### Imaging model: feature selection

**Fig. 4:**
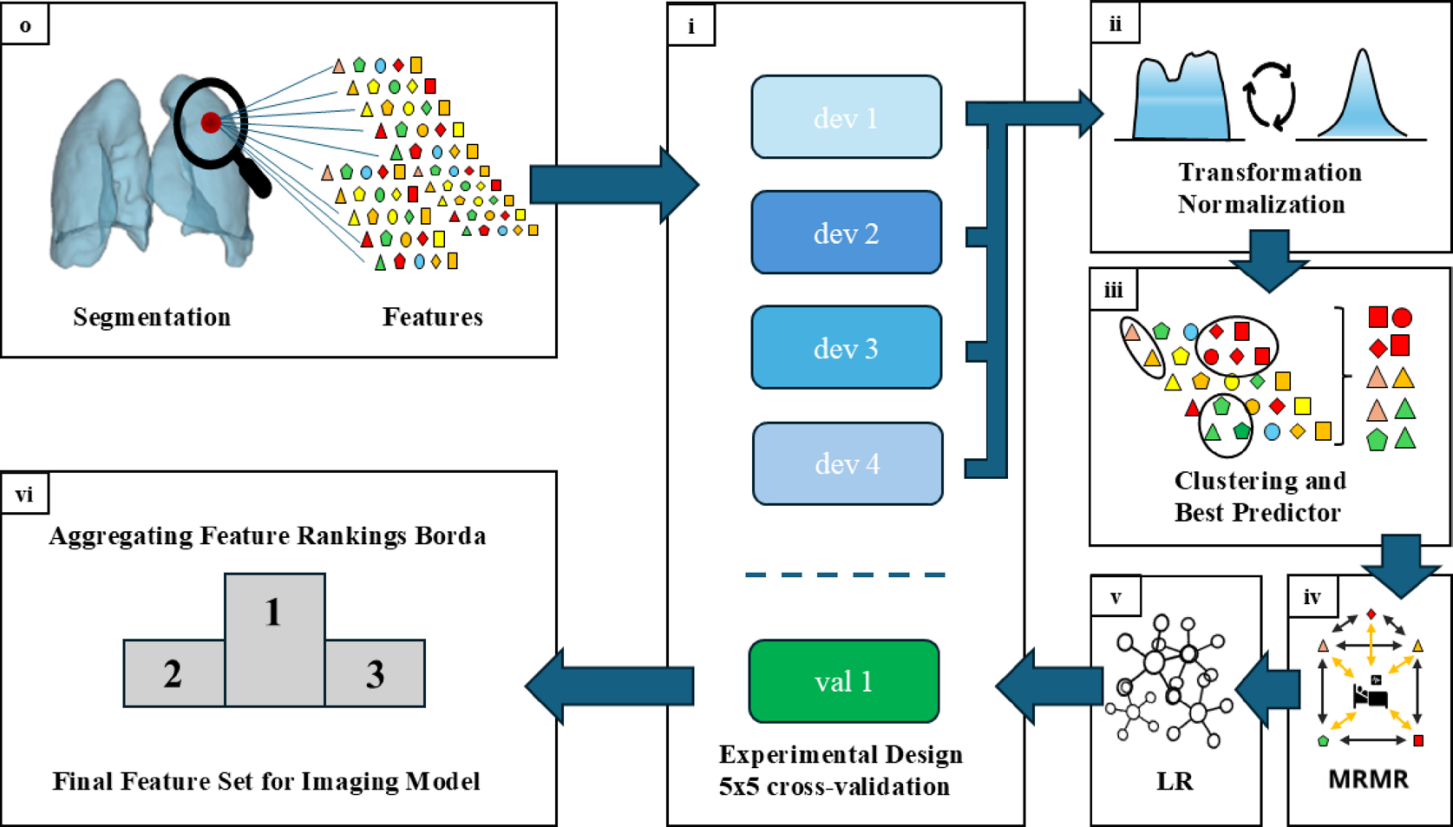
Overview of the machine learning pipeline for feature reduction of the imaging data. (**o**) CT-based Lung Segmentation and subsequent feature extraction, as described previously, yielded many imaging features. (**i**) The 5 × 5 cross-validation is the experimental setup. The reduction process is performed on each development fold (dev 1–4) and begins with (**ii**) Feature Standardization, including z-normalization and Yeo–Johnson transformation, followed by (**iii**) Feature Clustering and Exclusion using hierarchical agglomerative clustering and best predictor selection. Filtering with the Mann–Whitney U test was used for irrelevant feature exclusion. (**iv**) Feature Ranking is carried out via the MRMR algorithm to identify the most informative variables. This was followed by (**v**) Model development via logistic regression and internal validation on the validation fold (val) of the training cohort. (**vi**) Final Signature is defined by aggregating the feature rankings over all five cross-validation folds on the basis of the Borda score. MRMR: minimal redundancy maximum relevance; dev: development fold of the training cohort; val: validation fold of the training cohort.

Before training a machine learning model, it is essential to define and select the most informative features to avoid overfitting. The CT-derived imaging features consisted of a high-dimensional feature space with a total of 592 features per patient. The inclusion of all the features increases the risk of an overfitted model that may perfectly predict events in the training cohort, but owing to its tailoring to the training data, it may be poorly generalized to unseen cases. To address this, we used the R package “familiar”^20^ in RStudio (version 2024.04.1 + 748), which implements a configurable yet standardized pipeline for automated feature selection and model development. The process was conducted entirely within the training cohort and followed six core steps, which are illustrated in Fig. 4.

#### Cross-validation

A 5-times repeated 5-fold cross-validation strategy was employed for signature discovery. In each repetition, the training cohort was randomly divided into five folds: four used for model development (dev 1–4) and one for internal validation (val 1), such that every fold was used for internal validation once. Each development set underwent a feature reduction pipeline, beginning with feature standardization.

#### Feature standardization

The 592 imaging features from each development fold were first transformed via the Yeo‒Johnson transformation to better approximate a normal distribution^21^. The features were subsequently standardized via z-normalization, which adjusts the data such that each feature has a mean of 0 and a standard deviation of 1. Standardization is particularly beneficial in machine learning applications, as many algorithms are sensitive to the scale of the input variables.

#### Feature clustering and exclusion

To reduce the high-dimensional feature space, features were grouped via hierarchical agglomerative clustering (HAC) on the basis of Spearman correlation with a threshold of 0.75 and complete linkage. HAC is used to divide data into groups (clusters) on the basis of similarities. The main purpose is to discover structures in the data without having to know in advance how many clusters there are. For each cluster, the feature showing the highest univariate association with the outcome (ECMO) was selected to represent the cluster. Features with a p value > 0.2 in the Mann‒Whitney U test were excluded because they were considered irrelevant. This is a common approach in multistep selection processes where further filtering via MRMR ranking and collinearity checks is applied downstream.

#### Feature ranking

The remaining features were ranked by importance via the MRMR (Minimum Redundancy Maximum Relevance) algorithm^22^. This filter-based method prioritizes features that are maximally informative with respect to the outcome while being minimally redundant with each other, enabling the selection of a diverse and relevant subset. In image analysis, MRMR helps reduce the dimensionality of image data by prioritizing features that contribute most to the predictive model while avoiding overlap in the information they provide.

#### Model development

The remaining features were used for model development via logistic regression. The resulting model was validated on the respective internal validation fold.

#### Signature features

A final signature was subsequently defined by aggregating the feature rankings over all cross-validation experiments on the basis of the Borda score^23^ and selecting the three most relevant features of this ranking as candidates for the final model. The lower-ranked features showed a high degree of mutual correlation and were therefore filtered out. Within each rank, the most important feature was identified via a Wald test within logistic regression. Subsequently, features with a Spearman correlation > 0.5 and at least one more important feature were excluded to minimize redundancy.

### Clinical model: feature extraction and selection

To avoid overfitting and mutual correlation, only a small number of features were extracted. Clinical features were extracted on the basis of clinical evidence from the literature reflecting the severity of illness and correlations among these features. The selected features included *age*^24^, laboratory parameters (*lactate* [mmol/L] and *C-reactive protein* [mg/L]^25,26^, *positive end expiratory pressure (PEEP)* [cmH_2_O], and *mean airway pressure Pmean* [cmH_2_O]^27^. Feature selection was performed on the basis of correlation analysis of these extracted parameters to avoid mutual correlations among features.

### Model training and statistical analysis

Logistic regression was selected to develop all three models due to its interpretability (e.g., regression coefficients as odds ratios), its ability to directly estimate event probabilities, and its robustness in settings with moderately sized datasets. The developed models include (1) an Imaging Model that uses the final radiomic feature signature, (2) a Clinical Model that is based on selected clinical parameters, and (3) a Combined Model that integrates both feature sets into a single regression equation.

The final models were trained on the entire training cohort using the selected features. An optimal cutoff for risk prediction was determined via the Youden index^28^, which identifies the threshold that maximizes the sum of sensitivity and specificity. Model characteristics were described via regression coefficients, Wald statistics, and corresponding p values. Higher values of the Wald statistic indicate a more stable and reliable effect. The p value denotes the statistical significance of each variable, whereas larger regression coefficients indicate a stronger association with the vv-ECMO prediction within the model. Statistical analyses were performed via SPSS (Version 30.0.0.0 (IBM, INC, Armonk, NY, U.S.). The Kolmogorov–Smirnov test was used to assess the normality of continuous variables. Group comparisons were conducted via the Mann–Whitney U test for continuous variables and the chi-square test for categorical variables. Unless otherwise stated, continuous values are reported as the means ± standard deviations.

### Model validation

The final models and cutoffs were subsequently applied to the independent validation cohort. Model performance in predicting the need for vv-ECMO was assessed via chi-square statistics, sensitivity, specificity, positive predictive value, negative predictive value, and overall accuracy. In addition, the discriminative ability of each model was quantified by the area under the receiver operating characteristic curve (AUROC). To assess cumulative event differences, Kaplan–Meier curves with risk tables were generated, and statistical significance was evaluated via the log-rank test. To account for the possibility that patients could die before ECMO initiation, a competing risk analysis was applied^29^. Cumulative incidence functions (CIF) were estimated for ECMO initiation (i.e. event 1) and death without prior ECMO (i.e. event 2, the competing risk). Group differences in CIF curves were assessed using Gray’s test. In addition, subdistribution hazard ratios (SHR) with 95% confidence intervals were calculated using Fine–Gray proportional subdistribution hazards regression to quantify the association between model-predicted ECMO risk groups and the occurrence of each event while accounting for the competing risk. Gray’s test was chosen to evaluate overall differences in cumulative incidence between groups, whereas the Fine–Gray model allowed estimation of the effect size and statistical significance for the predefined covariate of interest. Both ECMO initiation and death without ECMO were analyzed separately to enable joint interpretation of the model’s discriminatory performance across endpoints.

## Results

### Characteristics of patients in the training and validation cohorts

**Fig. 5:**
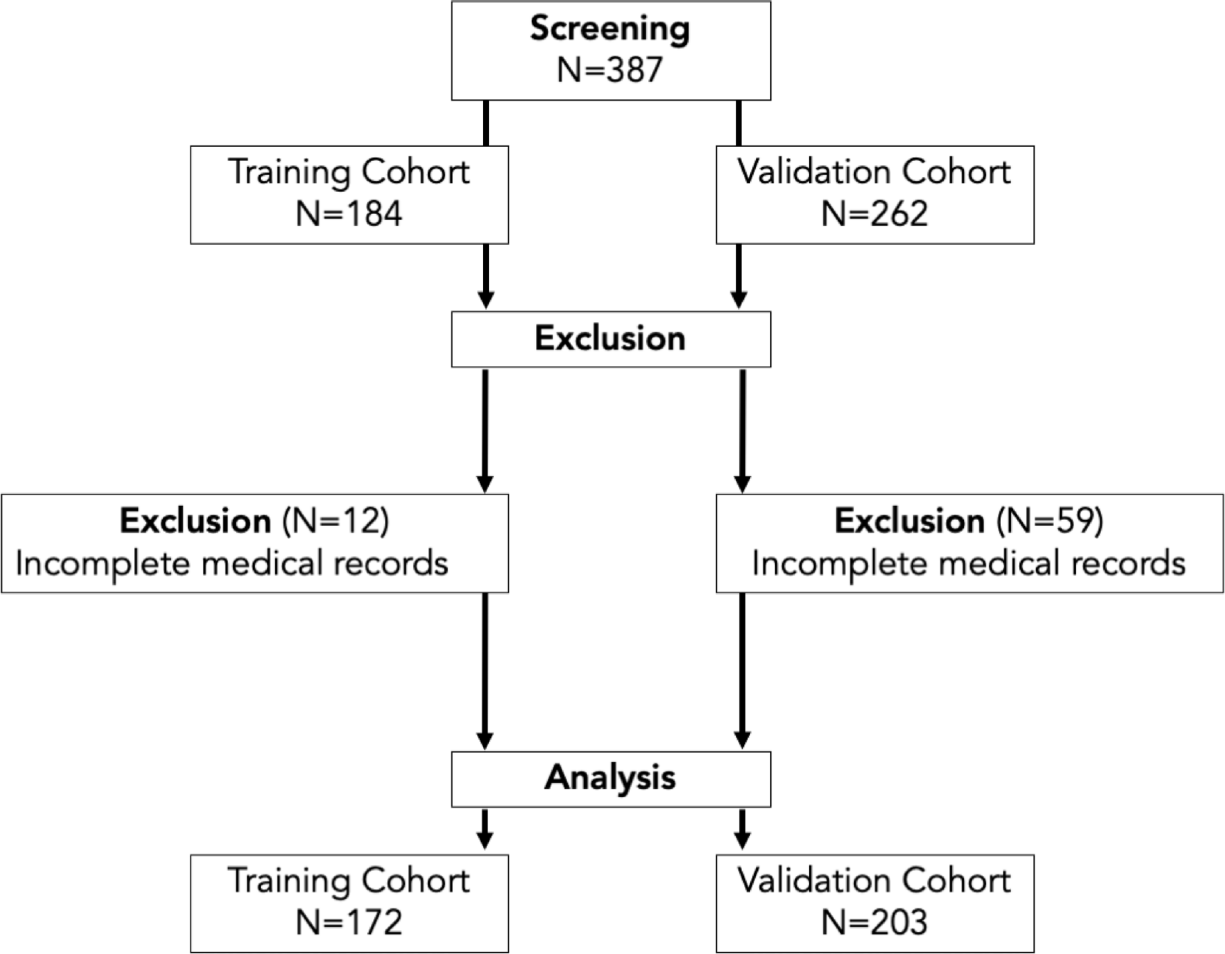
Study flow chart of patient inclusion. Retrospective patient screening was performed for patients admitted between March 2020 and March 2022. Patients who fulfilled the inclusion criteria and were admitted between March 19, 2020, and March 11, 2021, were allocated to the training cohort. Patients who fulfilled the inclusion criteria and were admitted to the ICU between March 11, 2021, and March 29, 2022 were allocated to the validation cohort.

**Fig. 6:**
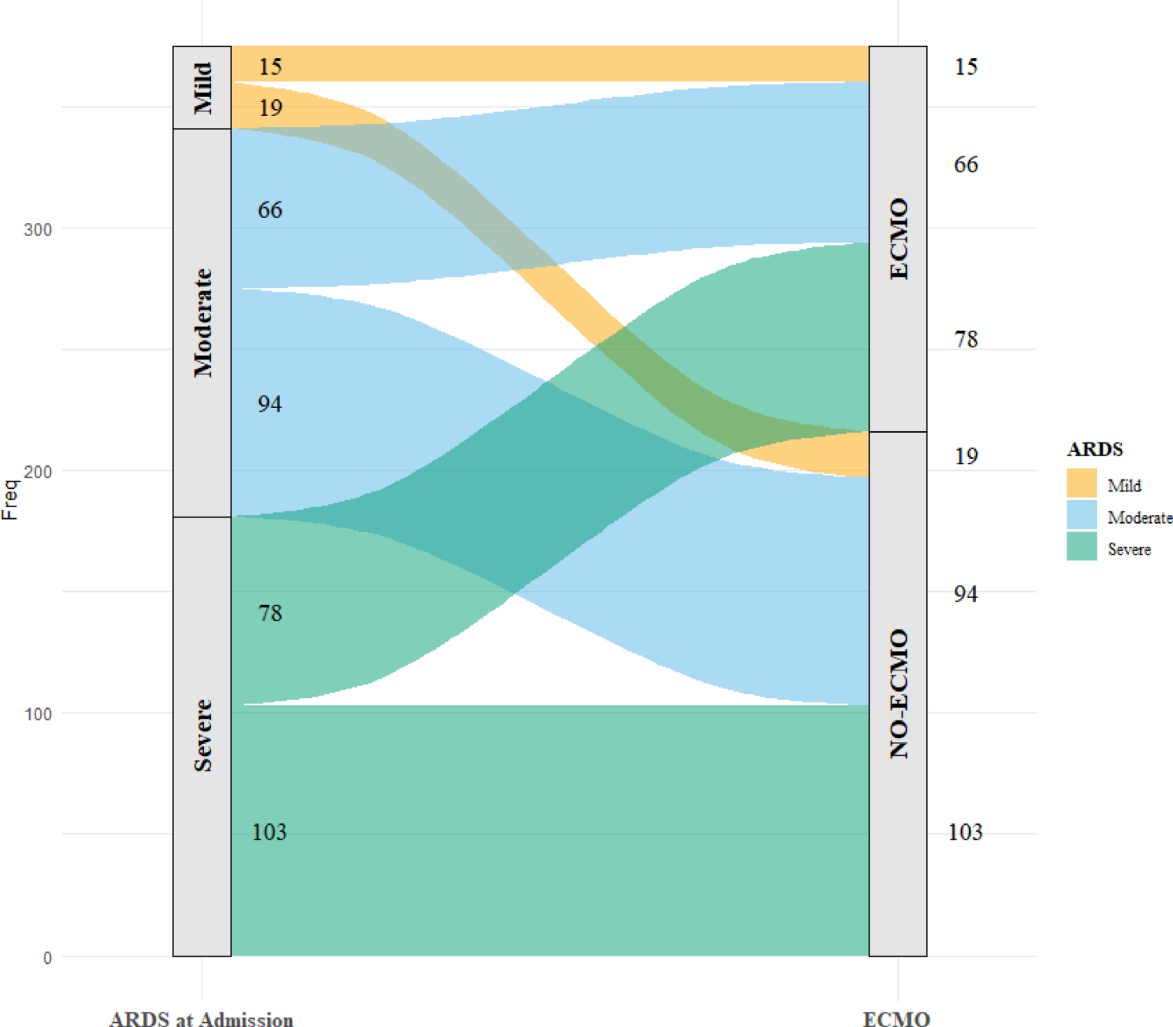
Distribution of patients receiving vv-ECMO according to ARDS severity at admission. Sankey diagram illustrating the distribution of vv-ECMO therapy according to ARDS severity at admission. Patients were categorized as having mild, moderate, or severe ARDS on the basis of established criteria. The width of each flow corresponds to the number of patients transitioning from each ARDS severity category to either vv-ECMO or no-ECMO treatment. Among patients with severe ARDS, the majority received vv-ECMO (55/103), whereas patients with mild ARDS were less likely to receive vv-ECMO therapy (5/19). The incidence of moderate ARDS was nearly equal between the vv-ECMO (44/81) and no-ECMO (37/81) groups. ARDS: Acute respiratory distress syndrome. Freq. : Absolute number of patients in each group.

After screening and exclusion, 172 patients in the training cohort and 203 patients in the validation cohort were available for analysis (Fig. 5). The patient characteristics of both cohorts are presented in Table 2. ECMO was required for 26.7% of the patients in the training cohort. At ICU admission, the prevalence of severe ARDS in the training cohort was 45.3%. Severe ARDS was present in 50.0% of patients who later required ECMO and in 43.7% of those who did not. In the validation cohort, 55.7% of all patients ultimately required ECMO. Severe ARDS was present in 50.7% of all patients and in 48.7% of those who subsequently required ECMO (Fig. 6).

**Table 2:** Characteristics of patients in the training and validation cohorts.

	Training		Validation		Training	Validation
	Non-ECMO (*n* = 126)	ECMO (*n* = 46)	Non-ECMO (*n* = 90)	ECMO (*n* = 113)	Full cohort (*n* = 172)	Full cohort (*n* = 203)
General information						
Age [years]	68.40 (9.53)	60.30 (8.70)**	60.83 (12.24)	55.40 (9.37)**	66.23 (9.96)	57.81 (11.05)**
Female Sex, n (%)	34 (27)	9 (20)	28 (31)	32 (28)	43 (25.0)	60 (29.6)
Weight [kg]	90.27 (20.75)	97.72 (20.59)*	94.46 (19.37)	98.47 (24.20)	92.26 (20.91)	96.69 (22.23)*
Body mass index [kg/m²]	29.51 (5.80)	32.35 (8.09)*	31.41 (6.16)	31.89 (7.14)	30.27 (6.58)	31.68 (6.71)*
Days in ICU	14.46 (9.78)	17.72 (11.28)	13.80 (8.64)	28.12 (19.01)**	15.33 (10.27)	21.77 (16.85)**
Mortality, n (%)	70 (55.6)	32 (69.6)	41 (45.6)	77 (68.1)*	102 (59.3)	118 (58.1)
Laboratory parameters						
C-reactive protein [mg/L]	158.4 (99.8)	218.5 (94.8)**	155.9 (109.9)	186.9 (116.1)	174.5 (101.8)	173.1 (114.1)
Blood lactate [mmol/L]	1.32 (0.77)	1.94 (1.71)*	1.72 (1.65)	1.74 (1.03)	1.48 (1.13)	1.73 (1.34)*
pH	7.38 (0.09)	7.39 (0.11)	7.35 (0.09)	7.38 (0.09)*	7.38 (0.10)	7.37 (0.09)
Course of disease						
CCI	4.33 (2.45)	2.54 (1.71)**	2.50 (1.97)	1.51 (1.20)**	3.85 (2.40)	1.95 (1.63)**
Hypertension, n (%)	94 (75)	26 (57)*	59 (66)	64 (57)	120 (69.8)	123 (60.6)
Diabetes, n (%)	59 (47)	14 (30)	28 (31)	27 (24)	73 (42.4)	55 (27.1)*
COPD, n (%)	9 (7)	3 (7)	2 (2)	2 (2)	12 (6.9)	4 (1.9)*
Pulmonary embolism, n (%)	38 (30)	19 (41)	44 (49)	74 (65)*	57 (33.1)	118 (58.1)**
ARDS mild, n (%)	14 (11)	1 (2)	5 (6)	13 (12)	15 (8.7)	18 (8.8)
ARDS moderate, n (%)	57 (45)	22 (48)	37 (41)	45 (40)	79 (45.9)	82 (40.4)
ARDS severe, n (%)	55 (44)	23 (5)	48 (53)	55 (49)	78 (45.3)	103 (50.7)
Respiratory parameters						
Days of ventilation	14.93 (9.71)	21.67 (11.34)**	14.26 (9.47)	31.97 (19.16)**	16.74 (10.57)	24.12 (17.91)**
Horovitz quotient [mmHg]	126.00 (59.71)	106.88 (40.74)	110.67 (49.78)	123.83 (66.71)	120.89 (55.81)	117.99 (60.02)
Driving pressure [cmH_2_O]	18.52 (2.91)	20.59 (3.10)*	19.94 (4.03)	21.60 (3.27)*	19.08 (3.91)	20.87 (3.71)**
Mean airway pressure [cmH_2_O]	12.63 (2.91)	14.00 (2.16)*	13.76 (2.83)	14.56 (2.40)*	13.00 (2.79)	14.20 (2.63)**

Patient characteristics at ICU admission. The first and second columns compare the characteristics at admission of patients who never required ECMO with those who did during their further clinical course, stratified by training and validation cohorts. The third column compares training and validation without distinguishing the need for ECMO. Statistically significant differences refer to comparisons within each column and are indicated by bold text and asterisks (*p < 0.05; **p < 0.001). The Mann‒Whitney U test was used for independent metric samples, and the chi‒square test was used for nominal values. Days of ventilation and mortality are linked to the entire course of illness, not just to ICU admission. Unless otherwise stated, values are presented as the means (standard deviations). ARDS: acute respiratory distress syndrome; CCI: Charlson comorbidity index; COPD: chronic obstructive lung disease; Days in the ICU: total days in the ICU; Horovitz: paO2/FiO2; ICU: intensive care unit; n.a.: data unavailable for non-ECMO patients; paO2: arterial partial pressure of oxygen; FiO2: fraction of inspired oxygen.

### Imaging model: feature selection, training, and performance

**Fig. 7:**
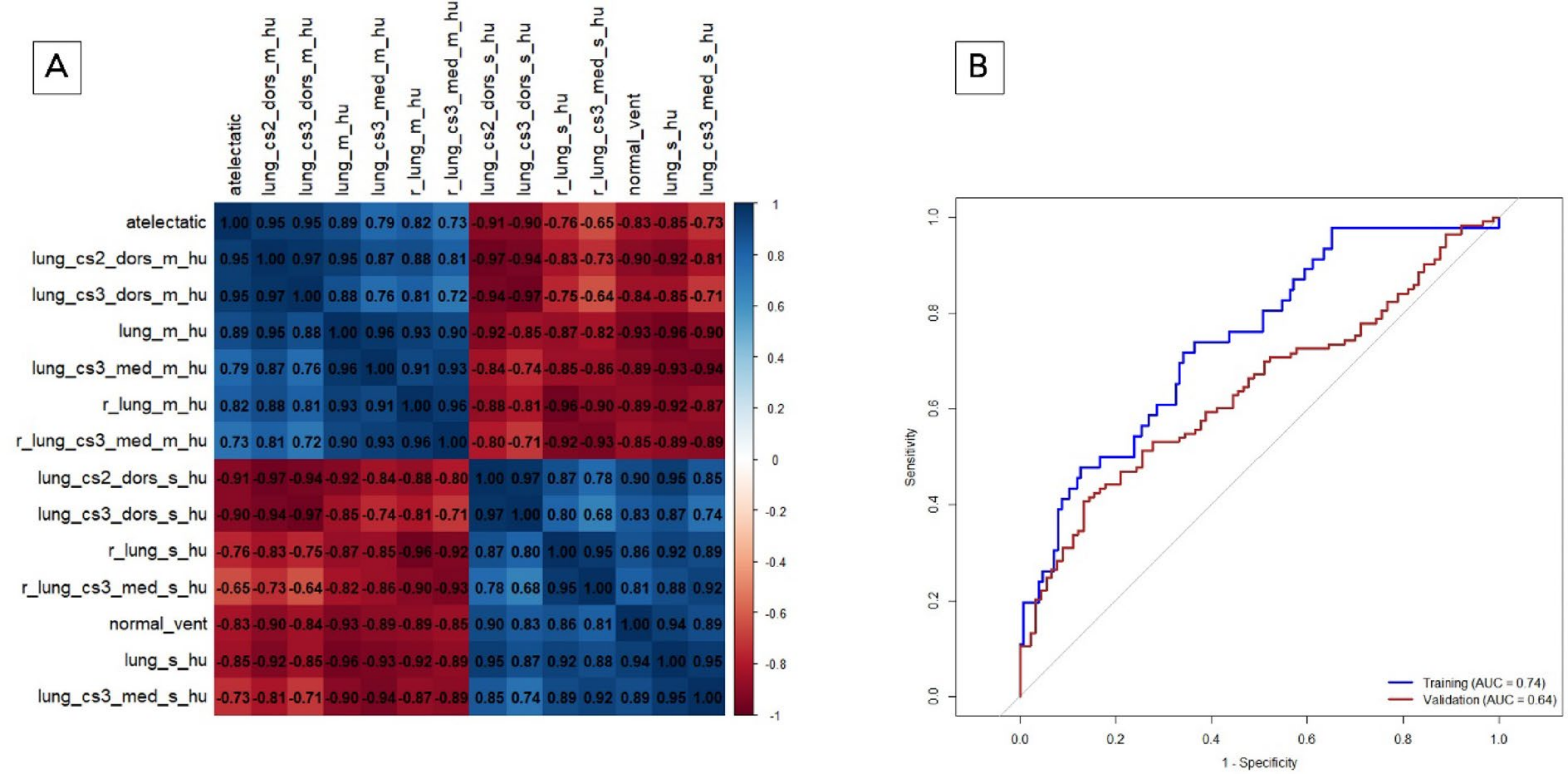
(**A**, **B**) Development and performance of the imaging model. (**A**) Correlation matrix for imaging features. Test of mutual correlation with Spearman correlation. The Spearman correlation coefficient is color-coded: blue indicates a positive correlation, whereas red represents a negative correlation. The intensity of the color reflects the strength of the correlation. The parameters are explained in Table S1 in the supplement. (**B**) Performance of the imaging model. The ROC curve for the Imaging Model in the training cohort (blue) had an AUROC of 0.743. The optimal threshold for the Imaging Model was determined via the Youden index, which yielded a value of 0.247. The performance of the Imaging Model in the validation cohort (red) resulted in an AUROC of 0.639.

Initial clustering, filtering, and cross-validation-based MRMR ranking yielded 14 potential model parameters with three different importance ranks, as presented in Table 3. The most influential imaging features for each rank group, based on Wald scores, were the proportion of normally aerated lung volume (norm_vent, rank 1), HU skew from the dorsal lung (lungs_cs2_dors_s_hu, rank 2) and the proportion of atelectatic lung volume (atelectatic, rank 3) (gray shaded in Table 3). These three image features exhibited high mutual Spearman correlations (Fig. 7A, Table S2). To prevent high collinearity of features in logistic regression, only one feature with the highest Wald statistic was selected. The final training model contains the “proportion of normally aerated lung volume” as the only imaging feature.

**Table 3:** Overview of the most prominent imaging features.

	Borda score	Rank	Wald score	*p* value	OR	95% CI	
						Lower	Upper
normal_ventilated [-]	14	1	22.123	< 0.001	0.002	0.000	0.027
lung_m_HU	14	1	20.666	< 0.001	1.007	1.004	1.010
r_lung_m_HU	14	1	19.850	< 0.001	1.007	1.004	1.009
lung_cs3_med_m_HU	14	1	18.799	< 0.001	1.007	1.004	1.010
lung_cs3_med_s_HU	14	1	18.428	< 0.001	0.238	0.124	0.458
lung_s_HU	14	1	18.147	< 0.001	0.214	0.105	0.435
r_lung_cs3_med_m_HU	14	1	16.867	< 0.001	1.006	1.003	1.008
r_lung_s_HU	14	1	16.601	< 0.001	0.280	0.152	0.517
r_lung_cs3_med_s_HU	14	1	16.489	< 0.001	0.321	0.186	0.556
lung_cs2_dors_s_HU	8	2	18.712	< 0.001	0.304	0.177	0.521
lung_cs3_dors_s_HU	8	2	17.424	< 0.001	0.378	0.240	0.597
Atelectatic [-]	7	3	21.475	< 0.001	1446.320	66.626	31396.825
lung_cs2_dors_m_HU	7	3	18.533	< 0.001	1.006	1.003	1.008
lung_cs3_dors_m_HU	7	3	15.665	< 0.001	1.005	1.002	1.007

Feature Selection for the Imaging Model. This table presents the feature rankings determined by the MRMR algorithm and the Borda score. Rank 1 represents the most influential feature in clustering parameters regarding ECMO therapy, followed by Rank 2 and Rank 3. The three most influential and robust parameters, identified by the highest Wald scores from logistic regression, were selected for further evaluation (gray shaded). All the parameters were significantly correlated with ECMO therapy (p value < 0.001). The odds ratio (OR) indicates the direction and magnitude of influence. For example, a high atelectatic volume is strongly correlated with the need for future ECMO (OR = 1446.3), whereas a high normally ventilated volume is strongly correlated with no need for future ECMO (OR = 0.002). The parameters are explained in Table S1 in the supplement.

CI: confidence interval; MRMR: minimum redundancy maximum relevance; OR: odds ratio; ECMO: extracorporeal membrane oxygenation; HU Hounsfield unit; CI: confidence interval.

The one-parameter Imaging Model is represented by the following formula:

$$\:P\left(ECMO=1|normal\_vent\right)=\frac{1}{1+{e}^{-\left(1.854+(-6.195*normal\_vent\right)}}$$

The strength of the feature’s influence in the Imaging Model is quantified by the Wald score and regression coefficient (Table 4). The Imaging Model yielded in 5-times repeated 5-fold cross-validation during model development, with an AUROC of 0.745 (95% CI: 0.629–0.850) for the respective internal validation folds in the training cohort. In the entire training cohort, the Imaging Model leads to an AUROC of 0.743 (95% CI: 0.661–0.826, *p* < 0.001) (Fig. 7B). For classification, a cutoff of 0.247 was selected by the Youden index on the training dataset.

In the validation cohort, the Imaging Model reached an AUROC of 0.639 (95% CI: 0.563–0.714, *p* < 0.001) (Fig. 7B). The Imaging Model predicted ECMO with an overall accuracy of 67.4% (*p* < 0.001) in the training cohort and 59.1% (*p* = 0.005) in the validation cohort (Tables 5, S3).

**Table 4:** Feature influence in imaging model.

	B	SE	Wald	*p* value	OR	95% CI for OR	
						Lower	Upper
Normal_vent [relative proportion]	-6.195	1.317	22.123	< 0.001	0.002	0.000	0.027

This table presents the strength of the feature’s influence quantified by the Wald score and regression coefficient. B: regression coefficient used for model development; SE: standard error; OR: odds ratio; CI: confidence interval; normal_vent: proportion of normal ventilated lung volume, expressed as a fraction of the overall volume of the lung.

**Table 5:** Performance of the prediction models in the training and validation cohorts.

	Imaging model		Clinical model			Combined model		
	Cohort		Cohort			Cohort		
	Training	Validation	Training		Validation	Training	Validation	
General information								
Observed need for ECMO	46	113	46		113	46	113	
Predicted need for ECMO	76	90	76		155	57	114	
Test metrics								
Sensitivity (%)	71.7	53.1	84.8		88.5	73.9	68.1	
Specificity (%)	65.9	66.7	70.6		38.9	81.8	58.9	
Positive predictive value (%)	43.4	66.7	51.3		64.5	59.7	67.5	
Negative predictive value (%)	86.5	53.1	92.7		72.9	89.6	59.6	
Overall accuracy (%)	67.4	59.1	74.4		66.5	79.7	64.0	

Performance metrics for all three models. The performances in the training and validation cohorts are depicted separately.

### Clinical model: feature selection, training, and performance

**Fig. 8:**
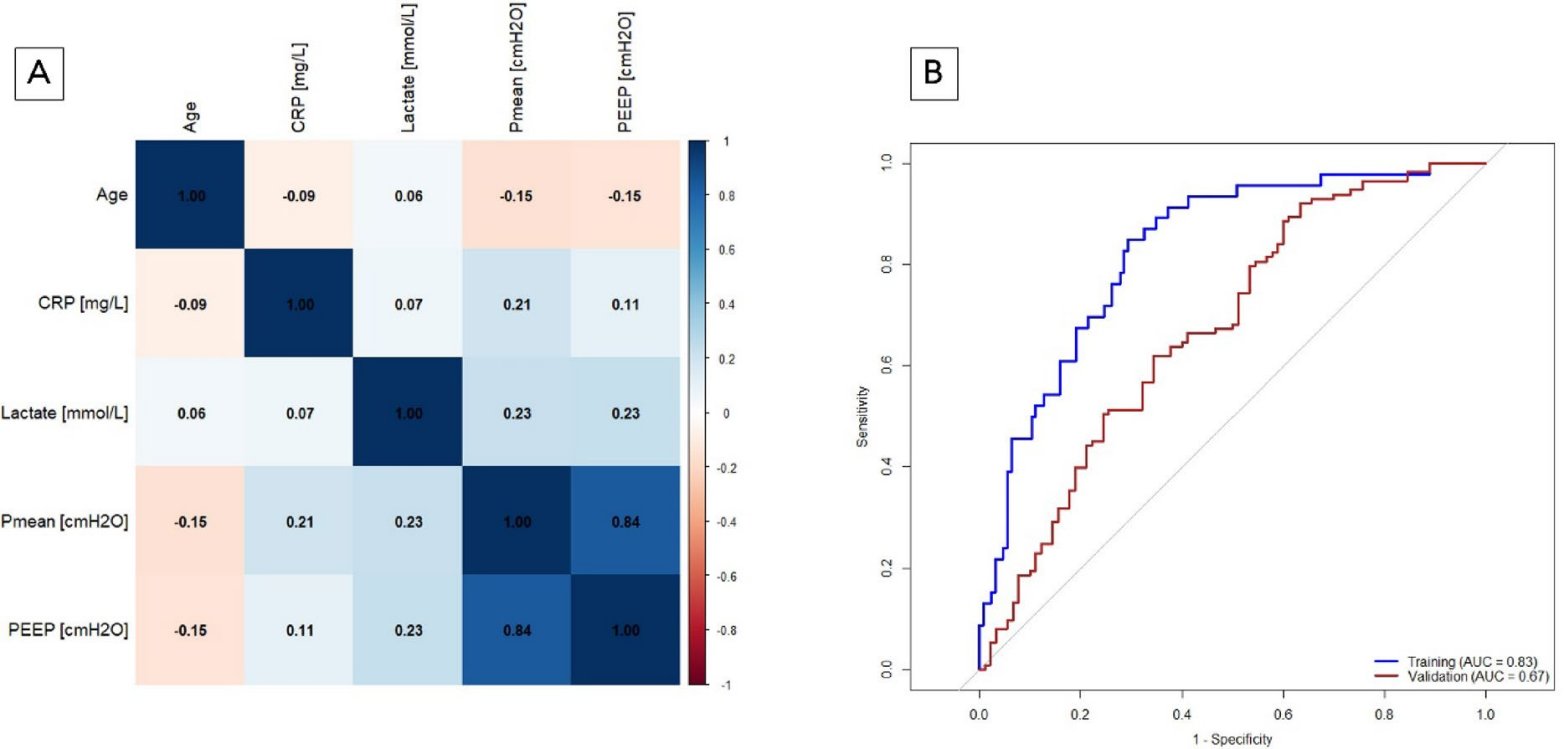
(**A**, **B**) Development and performance of the clinical model. (**A**) Correlation matrix for clinical features. Test of mutual correlation with Spearman correlation. The Spearman correlation coefficient is color-coded: blue indicates a positive correlation, whereas red represents a negative correlation. The intensity of the color reflects the strength of the correlation. CRP: C-reactive protein; Pmean: mean airway pressure; PEEP: positive end expiratory pressure. (**B**) Performance of the clinical model. The ROC curve for the Clinical Model in the training cohort (blue) had an AUROC of 0.828. The optimal threshold for the Clinical Model were determined via the Youden index, which yielded a value of 0.225. The performance of the Clinical Model in the validation cohort (red) resulted in an AUROC of 0.674.

The five selected clinical features were assessed for mutual correlation in the training cohort (Fig. 8A, Table S4). The two features PEEP and Pmean are highly correlated (r^2^ = 0.835, *p* < 0.001). To prevent high collinearity of features in logistic regression, of these two features, only one feature with the highest Wald statistic was selected (Table S5). The four remaining clinical features for the Clinical Model include: Age, Pmean, lactate level and CRP level at admission.

The four-parameter clinical-based model is represented by the following formula:

$$\:P\left(ECMO=1|Age,\:Pmean,\:Lactate,\:CRP\right)\:=\frac{1}{1+{e}^{-\left(1.594+(-0.095*Age+0.087*Pmean+0.559*Lactate+0.005*CRP\right)}}$$

The strength of the feature’s influence in the Imaging Model is quantified by the Wald score and regression coefficient (Table 6). In the training cohort, the clinical-based model led to an AUROC of 0.828 (95% CI: 0.762–0.894, *p* < 0.001) (Fig. 8B). For classification, a cutoff of 0.225 was selected by the Youden index on the training dataset.

**Table 6:** Feature influence in clinical model.

	B	SE	Wald	df	*p* value	OR	95% CI for OR	
							Lower	Upper
Age [years]	-0.095	0.023	17.207	1	< 0.001	0.910	0.870	0.951
Pmean [cmH_2_O]	0.087	0.057	2.335	1	0.126	1.091	0.976	1.220
Lactate [mmol/L]	0.559	0.225	6.154	1	0.013	1.749	1.125	2.721
CRP [mg/L]	0.005	0.002	6.153	1	0.013	1.005	1.001	1.009

This table presents the strength of the feature’s influence quantified by the Wald score and regression coefficient. CRP: C-reactive protein; B: regression coefficient, used for model development; SE: standard error; df: degree of freedom; OR: odds ratio; CI: confidence interval.

In the validation cohort, the Clinical Model had an AUROC of 0.674 (95% CI: 0.599–0.749, *p* < 0.001) (Fig. 8B). The Clinical Model predicted ECMO with an overall accuracy of 74.4% (*p* < 0.001) in the training cohort and 66.5% (*p* < 0.001) in the validation cohort (Table 5, S3).

### Combined model: feature selection, training, and performance

**Fig. 9:**
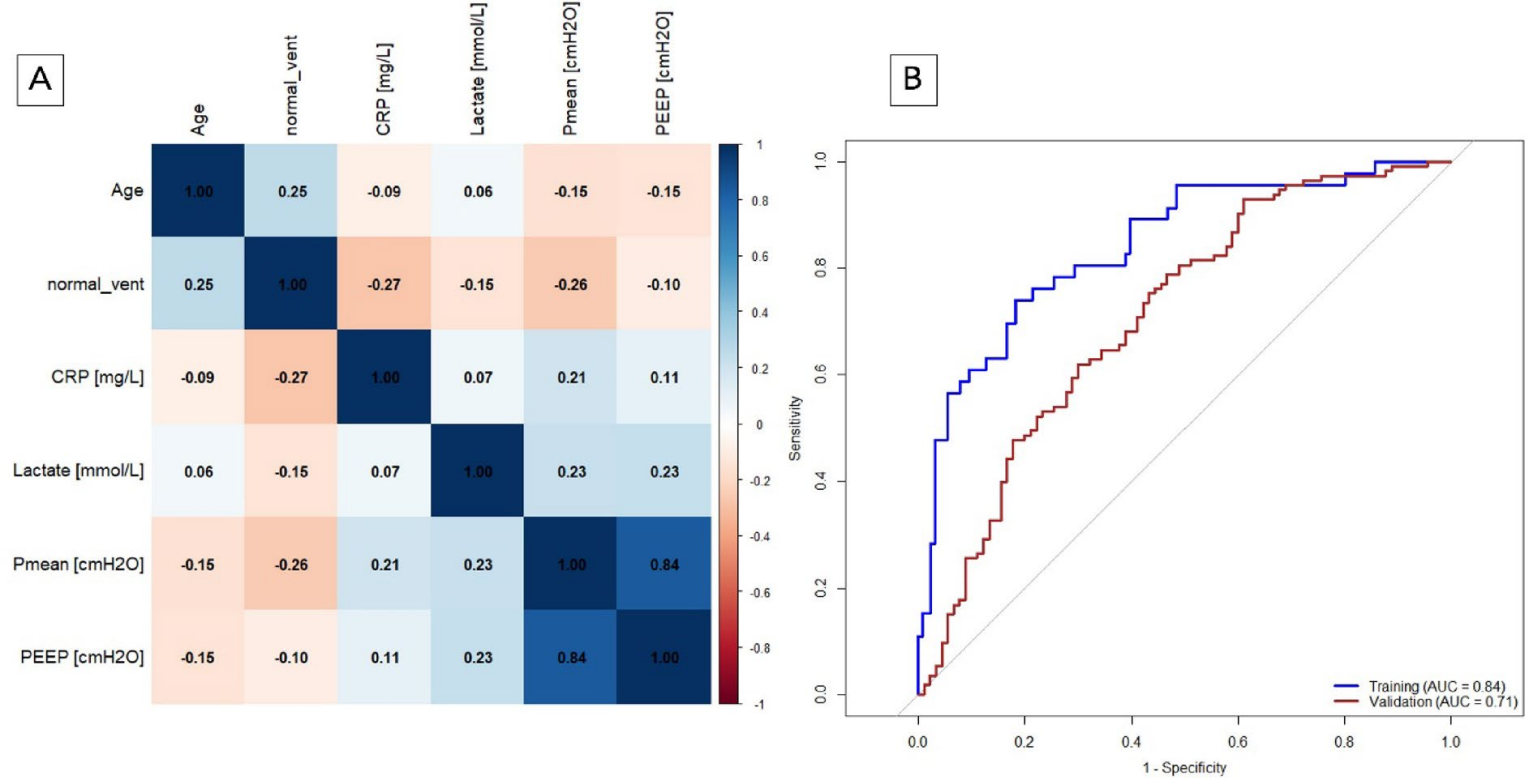
(**A**, **B**) Development and performance of the combined model. (**A**) Correlation matrix for combined features. Test of mutual correlation with Spearman. The Spearman correlation coefficient is color-coded: blue indicates a positive correlation, whereas red represents a negative correlation. The intensity of the color reflects the strength of the correlation. CRP: C-reactive protein; Pmean: mean airway pressure; PEEP: positive end expiratory pressure. (**B**) Performance of the combined model. The ROC curve for the Combined Model in the training cohort (blue) had an AUROC of 0.842. The optimal threshold for the combined model was determined via the Youden index, which yielded a value of 0.304. The performance of the Clinical Model in the validation cohort (red) resulted in an AUROC of 0.705.

For the Combined Model, features from both the imaging-based and the clinical-based models were selected. These five features were assessed for mutual correlation in the training cohort (Fig. 9A, Table S6). All five features were included in further model development because the highest absolute Spearman correlation coefficient was less than 0.5 (Fig. 9A, Table S6).

The five-parameter Combined Model is represented by the following formula:

$$\:P\left(ECMO=1|age,\:pmean,\:lactate,\:crp,norma{l}_{vent}\right)\:$$

$$\:=\frac{1}{1+{e}^{-\left(\text{3,775}+(-0.086*age+0.065*pmean+0.481*lactate+0.004*crp+\left(-4.396*normal\_vent\right))\right)}}$$

The strength of the feature’s influence in the imaging model is quantified by the Wald score and regression coefficient (Table 7). In the training cohort, the Combined Model leads to an AUROC of 0.842 (95% CI: 0.774–0.910, *p* < 0.001) (Fig. 9B). For classification, a cutoff of 0.304 was selected by the Youden index on the training dataset.

**Table 7:** Feature influence in combined model.

	B	SE	Wald	df	*p* value	OR	95% CI for OR	
							Lower	Upper
Age [years]	-0.086	0.024	13.183	1	< 0.001	0.917	0.0867	0.961
Pmean [cmH_2_O]	0.065	0.060	1.173	1	0.279	1.067	0.949	1.200
Lactate [mmol/L]	0.481	0.233	4.260	1	0.039	1.618	1.025	2.555
CRP [mg/L]	0.004	0.002	4.119	1	0.042	1.004	1.000	1.008
Normal_vent [relativ proportion]	-4.396	1.438	9.347	1	0.002	0.012	0.001	0.206

This table presents the strength of the feature’s influence quantified by the Wald score and regression coefficient. CRP: C-reactive protein; normal_vent: proportion of normal ventilated lung volume, expressed as a fraction of the overall volume of the lung; B: regression coefficient, used for model development; SE: standard error; df: degree of freedom; OR: odds ratio; CI: confidence interval.

In the validation cohort, the combined model had an AUROC of 0.705 (95% CI: 0.633–0.778, *p* < 0.001) (Fig. 9B). The Combined Model predicts vv-ECMO with an overall accuracy of 79.7% (*p* < 0.001) in the training cohort and 64.0% (*p* < 0.001) in the validation cohort (Table 5, S3).

### Model comparison: performance in the validation cohort

**Fig. 10:**
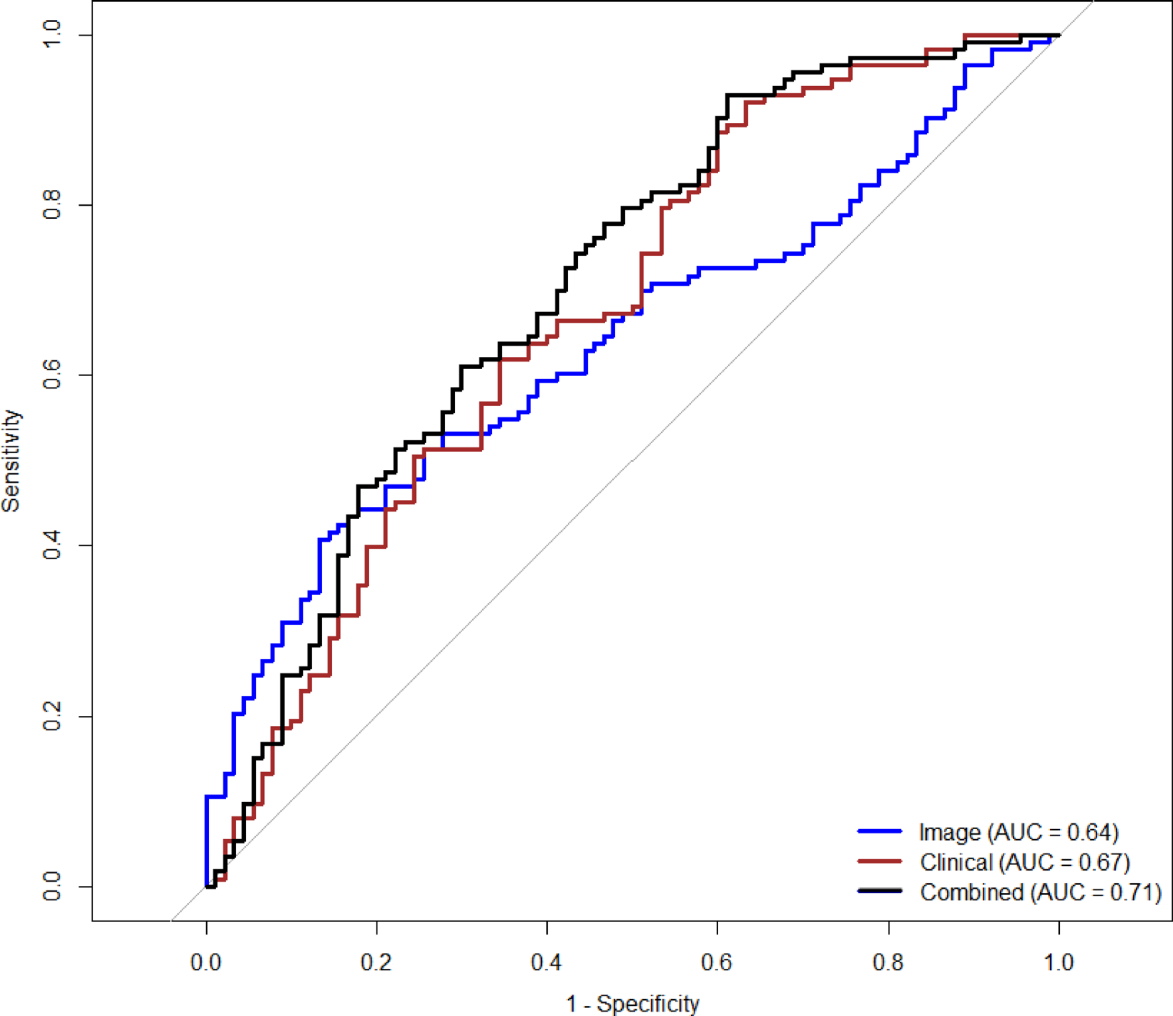
Comparison of model performance. The ROC curves for the validation cohort, comparing the predictive performance of the Imaging Model (blue), the Clinical Model (brown), and the Combined Model (black). The area under the curve (AUROC) was 0.64 for the Imaging Model, 0.67 for the Clinical Model, and 0.71 for the Combined Model.

**Fig. 11:**
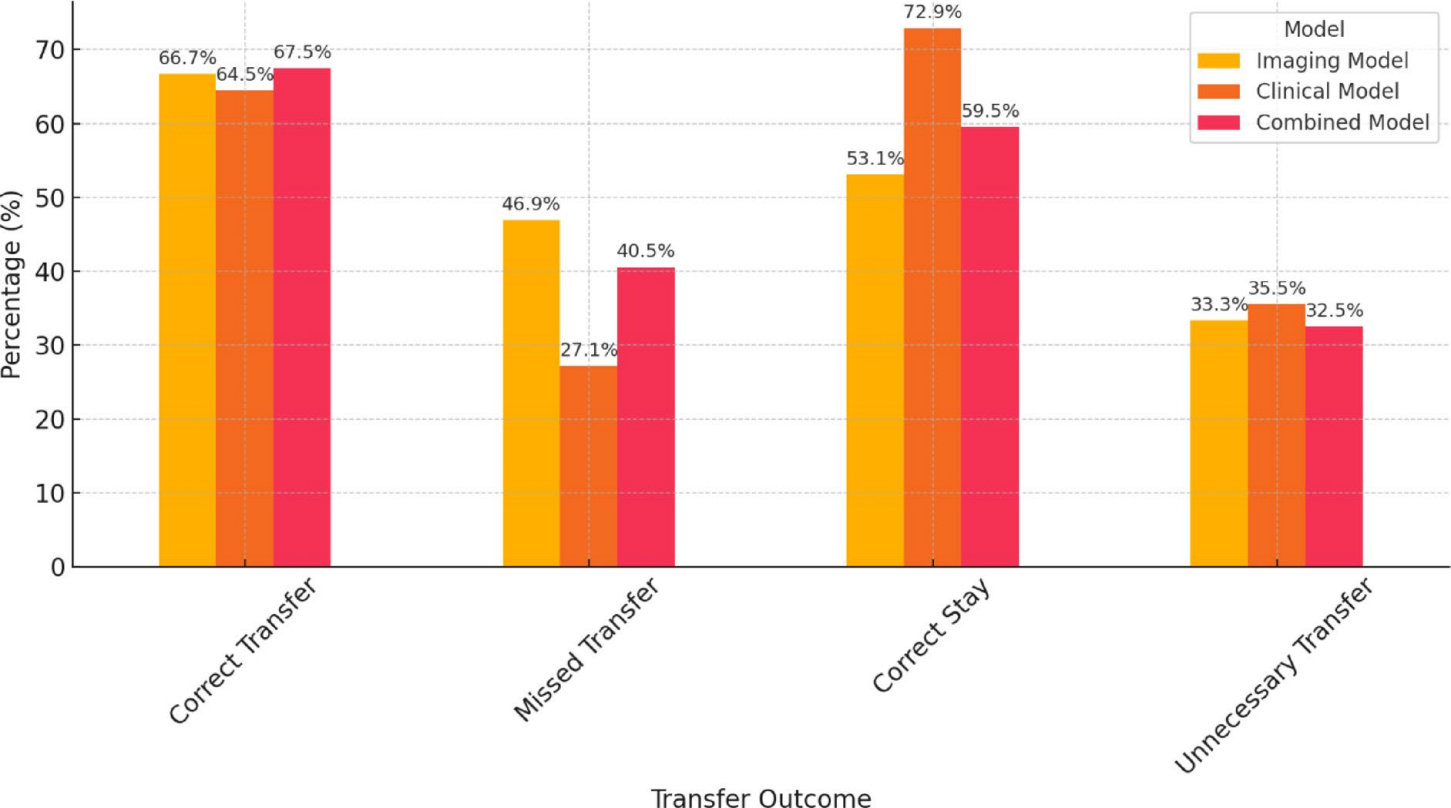
Outcomes in the validation cohort if the models guide transfer decisions. If ECMO prediction of the models would have led to transfer decisions, as in times of limited resources, transfer outcomes in the validation cohort would be as depicted in this figure. The x-axis groups predictions into four categories: correct transfer (true positives), missed transfer (false negatives), correct stay (true negatives), and unnecessary transfer (false positives). The percentages are shown for each outcome, with the Combined Model demonstrating the most balanced trade-off between avoiding unnecessary transfers and minimizing missed vv-ECMO cases.

**Fig. 12:**
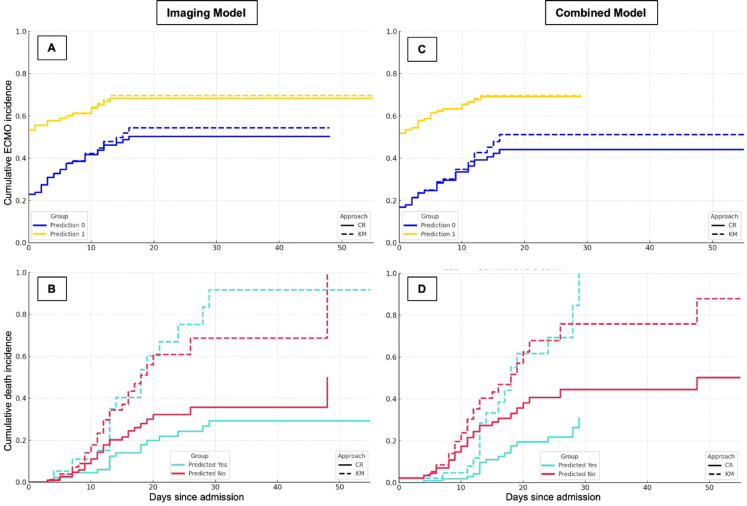
Cumulative incidence of ECMO initiation and death by prediction group: comparison of cumulative incidence function (CIF) and Kaplan-Meier (KM) estimates. (**A**, **C**) Cumulative incidence of ECMO initiation stratified by model prediction (predicted no-ECMO “0” vs. predicted ECMO “1”) for the Imaging Model (**A**) and the Combined Model (**C**). Solid lines represent CIF estimates accounting for death as a competing risk, whereas dashed lines indicate naive KM estimates treating competing events as censoring. Patients predicted to require ECMO (yellow) consistently showed higher ECMO incidence compared with those predicted not to require ECMO (blue). KM estimates tended to overestimate ECMO initiation probabilities relative to CIF. (**B**, **D**) Cumulative incidence of death stratified by model prediction for the Imaging Model (**B**) and the Combined Model (**D**). CIF estimates (solid lines) indicated higher death incidence in the predicted no-ECMO group (red), reflecting poorer outcomes in patients without ECMO therapy, while patients predicted to require ECMO (turquoise) demonstrated lower death incidence due to the competing risk of ECMO initiation. Naive KM estimates (dashed lines) underestimated mortality in the predicted no-ECMO group and overestimated it in the predicted ECMO group. CIF, cumulative incidence function; ECMO, CR, competing risk; extracorporeal membrane oxygenation; KM, Kaplan-Meier.

**Fig. 13:**
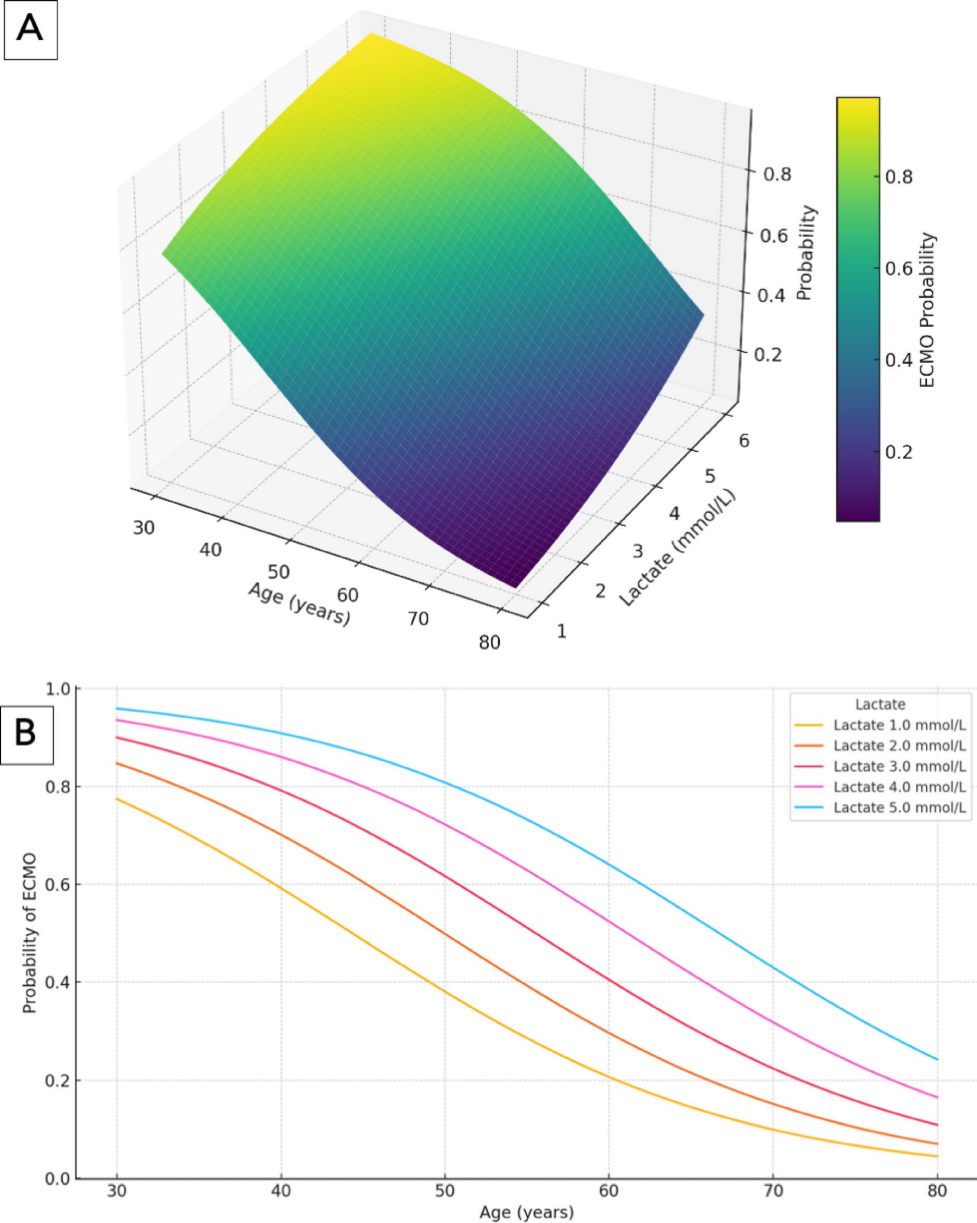
(**A**, **B**) Influence of age and lactate level on ECMO prediction in the combined model. (**A**) and (**B**) depict the ECMO prediction in the Combined as a function of age and lactate level, assuming that the CT feature “normal ventilation” = 0.492 and that the fixed values for CRP (196.0) and Pmean (14.4) correspond to the mean values for all patients within this study. (**A**) Three-dimensional surface plot showing the joint effect of age and lactate concentration on ECMO prediction. Younger age and higher lactate levels are associated with a greater likelihood of ECMO prediction in the Combined Model. (**B**) Two-dimensional plot illustrating ECMO prediction by age, stratified by lactate level (1.0–5.0 mmol/L). ECMO prediction decreases with increasing age across all lactate strata, with higher lactate levels consistently increasing the predicted probability.

Model performances in the validation cohort are summarized in Figs. 10, 11, 12, 13A,B, Tables 5, S3. The Combined Model demonstrated the highest discriminative ability (AUROC 0.71; overall accuracy 64.0%), outperforming both the Clinical Model (AUROC 0.67; 66.5%) and the Imaging Model (AUROC 0.64; 59.1%) (Fig. 10).

According to the *Imaging Model*, 90 of the 203 patients (44.3%) were classified as high risk for requiring ECMO therapy, yielding 60 true positives (66.7%) and 30 false positives (33.3%). Among the 113 patients predicted not to require ECMO therapy, 60 were true negatives (53.1%), and 53 were false negatives (46.9%) (Table 5). ECMO-free survival was significantly longer in the predicted no-ECMO group (24.5 ± 2.4 days vs. 18.0 ± 2.8 days; log-rank χ² = 9.856, *p* = 0.002) (Fig. 12). In the competing risk analysis, the Gray test confirmed a significant difference in the cumulative incidence of ECMO initiation between groups (*p* = 0.001), and Fine-Gray regression showed a 67% higher subdistribution hazard for ECMO in the “ECMO predicted” group (SHR = 1.67; 95% CI: 1.21–2.30; *p* = 0.002). For the competing event “death before ECMO,” no significant difference was observed (Gray test *p* = 0.071; Fine-Gray: SHR = 0.68; 95% CI: 0.38–1.20; *p* = 0.18).

Using the *Clinical Model*, 155 patients (76.4%) were classified as high risk for requiring ECMO therapy, with 100 true positives (64.5%) and 55 false positives (35.5%). Of the 48 patients predicted not to need ECMO, 35 were true negatives (72.9%), and 13 were false negatives (27.1%) (Table 5). ECMO-free survival was significantly longer in the no-ECMO group (36.9 ± 4.3 vs. 16.9 ± 1.8 days; log-rank χ² = 17.897, *p* < 0.001).

The *Combined Model* predicted 114 ECMO therapies (56.2%), with 77 true positives (67.5%) and 37 false positives (32.5%). Among the 89 patients who were predicted to be free of ECMO, 53 were true negatives (59.5%), and 36 were false negatives (40.5%). The ECMO-free time differed significantly between the groups (30.4 ± 3.1 vs. 9.9 ± 1.2 days; log-rank χ² = 16.923, *p* < 0.001) (Fig. 12). Competing risk analysis again demonstrated a significant difference in ECMO incidence between groups (Gray test *p* < 0.0001), with Fine-Gray regression indicating more than a doubling of the subdistribution hazard for ECMO in the “ECMO predicted” group (SHR = 2.11; 95% CI: 1.48–2.99; *p* < 0.0001). For “death before ECMO,” the cumulative incidence was significantly lower in the “ECMO predicted” group (Gray test *p* = 0.004), and Fine-Gray regression showed a 59% risk reduction (SHR = 0.41; 95% CI: 0.23–0.73; *p* = 0.002).

If the prediction models were used to guide transfer decisions from centers without ECMO capabilities or during times of limited resources, the Combined Model would offer the clearest temporal separation and the most balanced trade-off between overtriage and undertriage (Fig. 11).

The Kaplan-Meier curves and cumulative incidence function for ECMO-free survival stratified by model predictions are shown in Fig. 12.

A comparison of logistic regression performance and the area under the receiver operating characteristic curves for the three different models in the training cohort, as well as the corresponding AUROCs for the validation cohort, is provided in the supplementary material (Table S7).

To illustrate the magnitude of the influence of age and lactate on ECMO prediction in the Combined Model the quantitative CT feature “proportion of normal ventilation” was set at 0.492, corresponding to the mean of this parameter in both cohorts. Figure 13A,B illustrates the effects of age and lactate level on the ability of the Combined Model to predict ECMO. The 3D surface plot (Fig. 13A) revealed a strong interaction between age and lactate level: younger patients with elevated lactate levels presented the highest predicted ECMO probability. Conversely, the likelihood of ECMO decreases markedly with increasing age, particularly in patients with low lactate. This age-dependent decline is also evident in the 2D stratified plot (Fig. 13B), where the ECMO probability falls consistently across the age spectrum, whereas higher lactate values uniformly shift the probability curves upward. These findings underscore the synergistic contribution of metabolic and demographic factors to ECMO decision-making within the Combined Model.

## Discussion

This study presents three models to predict future vv-ECMO needs in ARDS patients, supporting timely, individualized therapy decisions while minimizing both overtreatment and delays in escalation. In scenarios of limited resources, objective prediction tools may also aid in determining whether a patient should be transferred to an ECMO center and help ECMO-capable institutions assess the urgency of accepting referred patients. The Combined Model developed in this study, which integrates clinical and imaging data at ICU admission, achieved a sensitivity of 68.1% and a specificity of 59.9% in the validation cohort. The Imaging Model reached a sensitivity of 53.1% and a specificity of 66.7%. The overall accuracy of the Combined Model achieved 64.0%.

Competing risk analysis showed that model-based classification was associated with clear differences in ECMO incidence, both in the Imaging and the Combined Model. The lower incidence of death without ECMO in the Combined Model group could reflect a survival benefit from early ECMO initiation in correctly identified high-risk patients; however, the retrospective design and differential mortality between groups may also inflate apparent discrimination by reducing ECMO incidence in the “no ECMO predicted” group. In the context of competing risks, higher early mortality can lead to seemingly greater separation between groups without necessarily improving identification of true ECMO candidates. Clinically, it is therefore essential to interpret ECMO initiation and death without ECMO jointly, and to assess whether patients in the “no ECMO predicted” group who died before ECMO might have been suitable candidates. Further analyses, such as cause-specific hazard models, are warranted to separate mortality effects from true predictive performance.

The ECMO course is often complicated by adverse events that are multifactorial and difficult to predict^30^. A timely individualized assessment of whether an ARDS patient is likely to progress toward ECMO dependence – independent of the overall prognosis – could support earlier and more targeted care pathways. This, in turn, might ultimately influence patient outcomes.

Numerous studies have evaluated survival outcomes in patients receiving vv- or va-ECMO^7,8^, often starting analysis immediately before cannulation, such as with the ECMOnet score^5^, or after cannulation^9^. However, only a few studies have attempted to predict the progression of ARDS toward the ECMO requirement at the time of ICU admission, when ECMO is not yet indicated^31^. While survival prediction following ECMO initiation is undoubtedly important, it remains questionable whether such prognostic estimates should influence the decision to initiate ECMO therapy. A timely, prognosis-independent assessment of the likelihood that an ARDS patient will deteriorate to the point of needing vv-ECMO may offer greater clinical utility. This is the first study to investigate the future need for vv-ECMO in patients with ARDS via models trained and validated on a temporally separated cohort. By integrating quantitative radiomic features derived from admission CT scans, the models developed in this study capture early and spatially resolved aspects of lung injury that are not readily apparent in conventional clinical scores. The radiomic features likely reflect pathophysiological hallmarks of severe ARDS, such as increased lung weight, consolidation, and fibroproliferative changes, which have previously been linked to recruitability and disease severity in CT-based studies by Gattinoni et al.^15^ and Protti et al.^16^. These structural alterations may explain the predictive association with later ECMO requirements, as also supported by the finding that traction bronchiectasis and mixed-density opacities are associated with prolonged ECMO duration and difficult liberation^9^. In the Imaging Model in this study, the ‘proportion of non-aerated lung areas’ emerged as a key feature in the final feature selection. This variable may reflect similar structural alterations as the ‘opacity’ characteristics identified by Nishikimi et al.^9^, particularly in terms of dense, non-ventilated parenchymal regions.

Our findings complement and extend prior work by Gresser et al.^31^, who demonstrated high predictive value for a combined SOFA score and CT-based lung involvement score in COVID-19 patients (AUC 0.91). In contrast to their approach, which focused on immediate hospital admission, our model begins at ICU admission and introduces feature-level granularity through automated radiomics. Additionally their approach did not include an independent validation cohort. The ‘lung involvement’ metric proposed by Gresser et al.^31^ resembles our selected feature ‘proportion of non-aerated lung areas’, as both quantify the extent of pulmonary abnormalities across the entire lung, providing a global perspective on disease burden. Compared with existing ECMO prediction tools such as the ECMOnet^5^ or RESP scores^6^, which rely primarily on clinical data shortly before cannulation, the method in this study enables earlier risk stratification and may support preemptive clinical decision-making.

Furthermore, radiomic CT features have demonstrated complementary value alongside clinical and laboratory markers in other models for predicting ICU admission, ventilation, or death in COVID-19 patients^32^. The added value of image-derived features suggests that quantitative CT analysis may provide important information on disease severity, distribution, and heterogeneity that traditional scoring systems fail to capture. Qualitative CT scan analysis and radiological findings alone are often insufficient to determine the need for ECMO therapy. A detailed quantitative analysis of lung CT scans based on machine learning models could provide additional insights; however, such analyses are currently not part of routine clinical practice owing to the labor-intensive nature of manual segmentation^33^. Automated tools leveraging modern computer vision and deep learning techniques such as the approach developed in this study could enable routine quantitative CT analysis and facilitate the development of novel decision-support systems.

The approach developed in this study could primarily support ECMO centers in making more timely and objective decisions regarding cannulation. Moreover, the model has the potential to facilitate structured, transparent dialog between referring institutions and ECMO centers, supporting coordinated transfer planning and optimal resource allocation. By enhancing the prediction of critical illness progression, healthcare resources can be allocated more efficiently, while patient safety can be improved through the early identification of clinical deterioration. This could facilitate earlier transfers of ECMO candidates while preventing unnecessary transfers of patients who do not require ECMO. A key advantage of the Combined Model in this study is the objectivity of the input criteria, making it independent of the treating physicians’ experience. At ICU admission, both patients who later required ECMO therapy and those who did not presented with moderate to severe ARDS. Therefore, the diagnosis of severe ARDS alone is not a reliable predictor of future ECMO requirements. Statistically significant differences were observed in the validation cohort between patients who required ECMO and those who did not regarding higher driving pressure and mean pressure, age, and comorbidities. However, the clinical relevance of these differences remains uncertain. Predicting ECMO necessity on the basis solely of these parameters is challenging, as it would require defining precise thresholds, which may vary across cohorts. Providing objective and early risk estimates contributes to more individualized treatment strategies and may help avoid both overtreatment and delayed initiation.

Given that timely decision-making is critical for patient survival^34^, such a model has the potential to significantly impact clinical outcomes. Nevertheless, further validation and a fully automated image segmentation approach are needed before deployment as a decision support tool for clinicians. In fact, current automatic preprocessing methods remain limited in handling certain pathological conditions, which may necessitate additional manual corrections and increase the overall time and effort required for data preparation.

### Model limitations and generalizability

While the Combined Model demonstrated solid predictive performance and supports individualized decision-making, several limitations must be emphasized. First, the clinical model includes only four parameters - age, lactate, mean airway pressure, and C-reactive protein - selected for their early availability, objectivity, and known relevance to disease severity. We acknowledge that this limited variable set cannot fully capture the clinical complexity of ARDS. Variables such as vasopressor use, lung compliance, and the P/F ratio may provide additional prognostic information but were either inconsistently documented or highly collinear with existing model components. Moreover, age and lactate are known factors in ECMO eligibility assessments and may reflect clinician bias or institutional practice patterns. This potential overlap highlights the risk of circular reasoning and underscores the need for further external validation in independent clinical settings.

Second, the study was conducted exclusively in COVID-19-associated ARDS patients retrospectively at a single center, which may limit generalizability to ARDS of other etiologies. This decision was based on the consistent availability of CT imaging and the high incidence of severe hypoxemic respiratory failure during the pandemic. However, we acknowledge that the pathophysiology and decision-making context for vv-ECMO may differ in bacterial pneumonia, trauma-related ARDS, or extrapulmonary etiologies. Future work should therefore evaluate the model’s performance in non-COVID ARDS populations. All patients were treated during the COVID-19 pandemic, a period characterized by dynamic resource constraints and altered clinical decision-making, particularly with respect to ECMO initiation, which may have influenced outcome distributions and model generalizability. However, all patients were treated at a single tertiary center, within a highly coordinated team, and under largely uniform decision-making standards at regarding ECMO initiation in our center. Although we deliberately validated the model in a temporally distinct cohort to test robustness, this temporal stratification may also reflect evolving treatment standards rather than pure differences in disease trajectory. As such, external validation across centres and time periods is essential before clinical deployment.

Third, although all patients met the Berlin criteria for ARDS and were admitted to a tertiary ICU with ECMO expertise, formal ECMO eligibility was not systematically documented at the time of admission. Some patients may have had predefined treatment limitations or comorbidities that would have precluded ECMO therapy, but these were not consistently captured in the dataset. Nevertheless, throughout the pandemic, the role of the ICU involved in this study remained constant: it served as the designated unit for patients with severe respiratory failure considered potential candidates for vv-ECMO. Admission to this unit therefore implicitly required the absence of formal contraindications to ECMO, even if this was not explicitly recorded. In addition, the institutional ECMO protocol mandates that patients transferred from external hospitals are accepted only if no known contraindications to ECMO exist - a requirement routinely confirmed prior to transfer. These factors suggest that, despite the absence of structured eligibility documentation, the cohort was composed of clinically appropriate ECMO candidates. However, potential selection bias cannot be entirely excluded and should be addressed in future prospective studies with clearly defined inclusion criteria. If such bias did occur, it would have likely affected both the training and validation cohorts in a similar and systematic manner. It should also be noted that, as a specialized tertiary care centre for ARDS and ECMO, a substantial proportion of patients were referred from external hospitals. This may have introduced referral and pretreatment bias. Furthermore, changes in clinical practice, triage policies, and resource availability between the early and later phases of the pandemic may have influenced the timing and frequency of ECMO initiation.

Fourth although CT scans are processed via a standardized CNN-based segmentation pipeline, manual review and correction of lung masks remain necessary, which may limit the feasibility of fully automated clinical implementation. Additionally, the lack of standardized CT acquisition protocols, e.g., the absence of respiratory-hold scanning and variability in ventilator settings between ward and transport ventilation, may have affected the consistency of the extracted radiomic features. Furthermore, clinical and imaging data are collected under routine clinical conditions and not always aligned temporally or methodologically; for example, ventilation parameters may differ slightly between the time of CT acquisition and concurrent clinical assessments. Although this introduces a degree of heterogeneity, it also enhances the real-world applicability of our findings. External validation in broader, multicenter ARDS cohorts and under standardized imaging protocols is therefore essential to confirm the robustness and clinical utility of the proposed model.

In contrast to end-to-end deep learning models, where feature selection is inherently embedded in the training process, our approach applies feature extraction and selection as separate steps. While this improves interpretability, it may limit the synergy between learned features and model optimization. Moreover, the logistic regression model, while transparent and easy to interpret, may not fully capture complex nonlinear relationships present in the data. Advanced models such as ensemble methods or deep learning architectures could offer improved predictive performance but were not explored here because of the focus on model explainability.

### Suggest the key takeaways from your paper

The automated analysis of chest-lung CT images has great potential for significantly reducing the time required to evaluate CTs in hospitals and thus contributes to faster therapy decisions. With the help of tools such as the Combined, the Clinical, and the Imaging Model, as proposed in this study, faster and safer decisions for or against ECMO therapy or referral to a center with ECMO capability can be made. Despite the imperfect standardized data collection, we were able to provide positive results for decision support and initial assessment. We see value in further investigations and data-driven and CT-based analyses to improve outcomes for patients with severe lung failure and the option of ECMO therapy. In this context, a prospective validation of the models developed in this study in non-COVID-19 ARDS patients would be valuable for assessing their generalizability.

## Conclusion

In this retrospective cohort study, we developed a machine learning-based model that integrates clinical parameters with quantitative imaging features derived from CT segmentation to predict the need for vv-ECMO therapy in patients with ARDS. The combined use of clinical and imaging data outperformed models based on clinical or imaging data alone, demonstrating improved accuracy in identifying patients at increased risk for ECMO. By providing an objective, data-driven tool for early risk stratification, this model may support clinicians in recognizing patients with a high likelihood of severe disease progression, thereby facilitating timely and individualized therapeutic decision-making in the management of ARDS.

## Supplementary Information

Below is the link to the electronic supplementary material.

Supplementary Material 1

## Data Availability

The datasets are not publicly available due to data sharing protocols but are available from the corresponding author upon reasonable request.

## Abbreviations

AI: Artificial intelligence
ARDS: Acute respiratory distress syndrome
AUROC: Area under the receiver operating characteristic curve
CNN: Convolutional neural network
COG: Center of gravity
CRP: C-reactive protein
CT: Computed tomography
DNN: Deep neural network
ECMO: Extracorporeal membrane oxygenation
HU: Hounsfield unit
ICU: Intensive care unit
ML: Machine learning
MRMR: Minimum redundancy maximum relevance
PEEP: Positive end-expiratory pressure
Pmean: Mean airway pressure
ROI: Region of interest
TRIPOD: Transparent reporting of a multivariable prediction model for individual prognosis or diagnosis
SHR: Subdistribution hazard ratio
va-ECMO: Veno-arterial extracorporeal membrane oxygenation
vv-ECMO: Veno-venous extracorporeal membrane oxygenation
