# Supplementary material for: Radiomics-enhanced modelling approach for predicting the need for ECMO in ARDS patients: a retrospective cohort study
Source: Sci Rep. 2025 Sep 30;15:34120. doi: 10.1038/s41598-025-21287-w (PMC12485099; doi:10.1038/s41598-025-21287-w)
Supplement: Supplementary file 1 — Supplementary Material 1 [file 41598_2025_21287_MOESM1_ESM.docx]

Radiomics-Enhanced Modelling Approach for Predicting the Need for ECMO in ARDS Patients: A Retrospective Cohort Study.

Martin Mirus, MD^1^; Eric Leitert^1^; Rebecca Bockholt, M.Sc.^1^; Lars Heubner, MD^1^; Steffen Löck, PhD^2^; Marie Brei, M.Sc.^3, 4^; Jonas Biehler, PhD^3, 4^; Jens-Peter Kühn, MD^5^; Thea Koch, MD^1^; Wolfgang Wall, PhD ^3^; Peter Markus Spieth, MD, M.Sc.^1*^

^1^ Department of Anaesthesiology and Intensive Care Medicine, Faculty of Medicine and University Hospital Carl Gustav Carus, TUD Dresden University of Technology, Fetscherstrasse 74, 01307 Dresden, Germany

^2^ OncoRay – National Center for Radiation Research in Oncology, Faculty of Medicine and University Hospital Carl Gustav Carus, TUD Dresden University of Technology, Fetscherstrasse 74, 01307 Dresden, Germany

^3^ Institute for Computational Mechanics, Technical University Munich, Boltzmannstraße 15, 85748 Munich, Germany

^4^ Ebenbuild GmbH, Holzstraße 28, 80469 Munich, Germany

^5^ Department of Diagnostic and Interventional Radiology, Faculty of Medicine and University Hospital Carl Gustav Carus, TUD Dresden University of Technology, Fetscherstrasse 74, 01307 Dresden, Germany

^*^ Corresponding author

**Supplement**

*Table S1: Spatial, functional, and intersectional regions of interest extracted by CT quantification within the lungs.*

| Parameter Abbreviation | Explanation |
| --- | --- |
| lung | lung |
| lung_cs2_vent | lung, coronal splits 2, ventral |
| lung_cs2_dors | lung, coronal splits 2, dorsal |
| lung_cs3_vent | lung, coronal splits 3, ventral |
| lung_cs3_med | lung, coronal splits 3, medial |
| lung_cs3_dors | lung, coronal splits 3, dorsal |
| l_lung | left lung |
| l_lung_cs2_vent | left lung, coronal splits 2, ventral |
| l_lung_cs2_dors | left lung, coronal splits 2, dorsal |
| l_lung_cs3_vent | left lung, coronal splits 3, ventral |
| l_lung_cs3_med | left lung, coronal splits 3, medial |
| l_lung_cs3_dors | left lung, coronal splits 3, dorsal |
| r_lung | right lung |
| r_lung_cs2_vent | right lung, coronal splits 2, ventral |
| r_lung_cs2_dors | right lung, coronal splits 2, dorsal |
| r_lung_cs3_vent | right lung, coronal splits 3, ventral |
| r_lung_cs3_med | right lung, coronal splits 3, medial |
| r_lung_cs3_dors | right lung, coronal splits 3, dorsal |
| p_lung | lung poorly ventilated |
| p_lung_cs2_vent | lung poorly ventilated, coronal splits 2, ventral |
| p_lung_cs2_dors | lung poorly ventilated, coronal splits 2, dorsal |
| p_lung_cs3_vent | lung poorly ventilated, coronal splits 3, ventral |
| p_lung_cs3_med | lung poorly ventilated, coronal splits 3, medial |
| p_lung_cs3_dors | lung poorly ventilated, coronal splits 3, dorsal |
| a_lung | lung atelectatic |
| a_lung_cs2_vent | lung atelectatic, coronal splits 2, ventral |
| a_lung_cs2_dors | lung atelectatic, coronal splits 2, dorsal |
| a_lung_cs3_vent | lung atelectatic, coronal splits 3, ventral |
| a_lung_cs3_med | lung atelectatic, coronal splits 3, medial |
| a_lung_cs3_dors | lung atelectatic, coronal splits 3, dorsal |
| nv_lung | lung normally ventilated |
| nv_lung_cs2_vent | lung normally ventilated, coronal splits 2, ventral |
| nv_lung_cs2_dors | lung normally ventilated, coronal splits 2, dorsal |
| nv_lung_cs3_vent | lung normally ventilated, coronal splits 3, ventral |
| nv_lung_cs3_med | lung normally ventilated, coronal splits 3, medial |
| nv_lung_cs3_dors | lung normally ventilated, coronal splits 3, dorsal |
| o_lung | lung overinflated |
| o_lung_cs2_vent | lung overinflated, coronal splits 2, ventral |
| o_lung_cs2_dors | lung overinflated, coronal splits 2, dorsal |
| o_lung_cs3_vent | lung overinflated, coronal splits 3, ventral |
| o_lung_cs3_med | lung overinflated, coronal splits 3, medial |
| o_lung_cs3_dors | lung overinflated, coronal splits 3, dorsal |

*Table S2 Imaging Model: Spearman correlation of the three most relevant imaging features in the training cohort.*

|  | Spearman's rho | Significance  (2-tailed) | 95% Confidence Intervals  (2-tailed)^a,b^ | |
| --- | --- | --- | --- | --- |
|  |  |  | Lower | Upper |
| normal_vent -  lung_cs2_dors _s_hu | 0.896 | <.001 | 0.861 | 0.923 |
| normal_vent - atelectatic | -0.830 | <.001 | -0.872 | -0.775 |
| lung_cs2_dors _s_hu - atelectatic | -0.909 | <.001 | -0.932 | -0.877 |
| Spearman-Rho correlation between three imaging features.  a. Estimation is based on Fisher's r-to-z transformation. | | | | |
| b. Estimation of standard error is based on the formula proposed by Fieller, Hartley, and Pearson. | | | | |

*Table S3 Confusion matrix for the training cohort and validation cohort (Imaging, Clinical, and Combined Model).*

| Imaging | Cutoff | **Training**  **(ECMO)** | Prediction | | |  | | Overall Accuracy | Chi² | p Value |
| --- | --- | --- | --- | --- | --- | --- | --- | --- | --- | --- |
|  | 0.247 | ground truth | *no* | *yes* | *all* | Sensitivity | Specificity | 0.674 | 19.33 | <0.001 |
|  |  | *no* | 83 | 43 | 126 | 0.717 | 0.659 |  |  |  |
|  |  | *yes* | 13 | 33 | 46 | PPV | NPV |  |  |  |
|  |  | *all* | 96 | 76 | 172 | 0.434 | 0.865 |  |  |  |
| Clinical | Cutoff | **Training**  **(ECMO)** | Prediction | | |  | |  | Chi² |  |
|  | 0.225 | ground truth | *no* | *yes* | *all* | Sensitivity | Specificity | 0.744 | 41.94 | <0.001 |
|  |  | *no* | 89 | 37 | 126 | 0.848 | 0.706 |  |  |  |
|  |  | *yes* | 7 | 39 | 46 | PPV | NPV |  |  |  |
|  |  | *all* | 96 | 76 | 172 | 0.513 | 0.927 |  |  |  |
| Combined | Cutoff | **Training**  **(ECMO)** | Prediction | | |  | |  | Chi² |  |
|  | 0.304 | ground truth | *no* | *yes* | *all* | Sensitivity | Specificity | 0.797 | 47.12 | <0.001 |
|  |  | *no* | 103 | 23 | 126 | 0.739 | 0.818 |  |  |  |
|  |  | *yes* | 12 | 34 | 46 | PPV | NPV |  |  |  |
|  |  | *all* | 115 | 57 | 172 | 0.597 | 0.896 |  |  |  |
| Imaging | Cutoff | **Validation**  **(ECMO)** | Prediction | | |  | | Overall Accuracy | Chi² | p Value |
|  | 0.247 | ground truth | *no* | *yes* | *all* | Sensitivity | Specificity | 0.591 | 7.93 | 0.005 |
|  |  | *no* | 60 | 30 | 90 | 0.531 | 0.667 |  |  |  |
|  |  | *yes* | 53 | 60 | 113 | PPV | NPV |  |  |  |
|  |  | *all* | 113 | 90 | 203 | 0.667 | 0.531 |  |  |  |
| Clinical | Cutoff | **Validation**  **(ECMO)** | Prediction | | |  | |  | Chi² |  |
|  | 0.225 | ground truth | *no* | *yes* | *all* | Sensitivity | Specificity | 0.665 | 20.81 | <0.001 |
|  |  | *no* | 35 | 55 | 90 | 0.885 | 0.389 |  |  |  |
|  |  | *yes* | 13 | 100 | 113 | PPV | NPV |  |  |  |
|  |  | *all* | 48 | 155 | 203 | 0.645 | 0.729 |  |  |  |
| Combined | Cutoff | **Validation**  **(ECMO)** | Prediction | | |  | |  | Chi² |  |
|  | 0.304 | ground truth | *no* | *yes* | *all* | Sensitivity | Specificity | 0.640 | 14.87 | <0.001 |
|  |  | *no* | 53 | 37 | 90 | 0.681 | 0.589 |  |  |  |
|  |  | *yes* | 36 | 77 | 113 | PPV | NPV |  |  |  |
|  |  | *all* | 89 | 114 | 203 | 0.675 | 0.596 |  |  |  |

*Table S4 Clinical Model: Spearman correlation of clinical features in the training cohort.*

|  | Spearman's rho | Significance  (2-tailed) | 95% Confidence Intervals  (2-tailed)^a,b^ | |
| --- | --- | --- | --- | --- |
|  |  |  | Lower | Upper |
| Age - Pmean | -0.150 | 0.049 | -0.298 | 0.004 |
| Age - PEEP | -0.148 | 0.052 | -0.295 | 0.006 |
| Age - Lactate | 0.056 | 0.467 | -0.099 | 0.208 |
| Age - CRP | -0.131 | 0.087 | -0.279 | 0.024 |
| Pmean - PEEP | 0.835 | <0.001 | 0.782 | 0.877 |
| Pmean - Lactate | 0.228 | 0.003 | 0.077 | 0.369 |
| Pmean - CRP | 0.080 | 0.298 | -0.075 | 0.231 |
| PEEP - Lactate | 0.232 | 0.002 | 0.081 | 0.373 |
| PEEP - CRP | 0.054 | 0.480 | -0.101 | 0.206 |
| Lactate - CRP | 0.008 | 0.916 | -0.146 | 0.162 |
| a. Estimation is based on Fisher's r-to-z transformation. | | | | |
| b. Estimation of standard error is based on the formula proposed by Fieller, Hartley, and Pearson. | | | | |

*Table S5: Overview of the most prominent clinical features.*

|  | | B | SE | Wald | Sig. | OR | 95% CI for OR | |
| --- | --- | --- | --- | --- | --- | --- | --- | --- |
|  |  |  |  |  |  |  | Lower | Upper |
|  | PEEP | 0.198 | 0.071 | 7.688 | 0.006 | 1.219 | 1.060 | 1.403 |
|  | Pmean | 0.156 | 0.053 | 8.836 | 0.003 | 1.169 | 1.055 | 1.296 |

*Table S6: Combined Model: Spearman correlation of combined features in the training cohort.*

|  | Spearman's rho | Significance  (2-tailed) | 95% Confidence Intervals  (2-tailed)^a,b^ | |
| --- | --- | --- | --- | --- |
|  |  |  | Lower | Upper |
| Age - Pmean | -0.150 | 0.049 | -0.298 | 0.004 |
| Age - PEEP | -0.148 | 0.052 | -0.295 | 0.006 |
| Age - Lactate | 0.056 | 0.467 | -0.099 | 0.208 |
| Age - CRP | -0.131 | 0.087 | -0.279 | 0.024 |
| Age - normal_vent | 0.252 | <0.001 | 0.102 | 0.391 |
| Pmean - PEEP | 0.835 | <0.001 | 0.782 | 0.877 |
| Pmean - Lactate | 0.228 | 0.003 | 0.077 | 0.369 |
| Pmean - CRP | 0.080 | 0.298 | -0.075 | 0.231 |
| Pmean - normal_vent | -0.259 | <0.001 | -0.397 | -0.110 |
| PEEP - Lactate | 0.232 | 0.002 | 0.081 | 0.373 |
| PEEP - CRP | 0.054 | 0.480 | -0.101 | 0.206 |
| PEEP - normal_vent | -0.098 | 0.203 | -0.248 | 0.057 |
| Lactate - CRP | 0.008 | 0.916 | -0.146 | 0.162 |
| Lactate - normal_vent | -0.149 | 0.052 | -0.296 | 0.006 |
| CRP - normal_vent | -0.160 | 0.036 | -0.306 | -0.006 |
| a. Estimation is based on Fisher's r-to-z transformation. | | | | |
| b. Estimation of standard error is based on the formula proposed by Fieller, Hartley, and Pearson. | | | | |

*Table S7: Conclusion from regression and AUROC results for the training and validation cohorts (Imaging, Clinical, and Combined Model).*

| Model  (training cohort, n=172) | Feature | OR (95% CI) | *p* value | AUROC (95% CI) | *p* value |
| --- | --- | --- | --- | --- | --- |
| Imaging | normal_vent | 0.002 (0.000 - 0.027) | <0.001 | 0.743 (0.661 - 0.826) | <0.001 |
| Clinical | Age | 0.910 (0.870 - 0.951) | <0.001 | 0.828 (0.762 - 0.894) | <0.001 |
|  | Pmean | 1.091 (0.976 - 1.220) | 0.126 |  |  |
|  | Lactate | 1.749 (1.125 - 2.721) | 0.013 |  |  |
|  | CRP | 1.005 (1.001 - 1.009) | 0.013 |  |  |
| Combined | Age | 0.917 (0.876 - 0.961) | <0.001 | 0.842 (0.774 - 0.910) | <0.001 |
|  | Pmean | 1.067 (0.949 - 1.200) | 0.279 |  |  |
|  | Lactate | 1.618 (1.025 - 2.555) | 0.039 |  |  |
|  | CRP | 1.004 (1.000 - 1.008) | 0.042 |  |  |
|  | normal_vent | 0.012 (0.001 - 0.206) | 0.002 |  |  |

| Model  (validation cohort, n=203) | Feature | AUROC (95% CI) | *p* value |
| --- | --- | --- | --- |
| Imaging | normal_vent | 0.639 (0.563 - 0.714) | <0.001 |
| Clinical | Age | 0.674 (0.599 - 0.749) | <0.001 |
|  | Pmean |  |  |
|  | Lactat |  |  |
|  | CRP |  |  |
| Combined | Age | 0.705 (0.633 - 0.778) | <0.001 |
|  | Pmean |  |  |
|  | Lactate |  |  |
|  | CRP |  |  |
|  | Normal_vent |  |  |

*
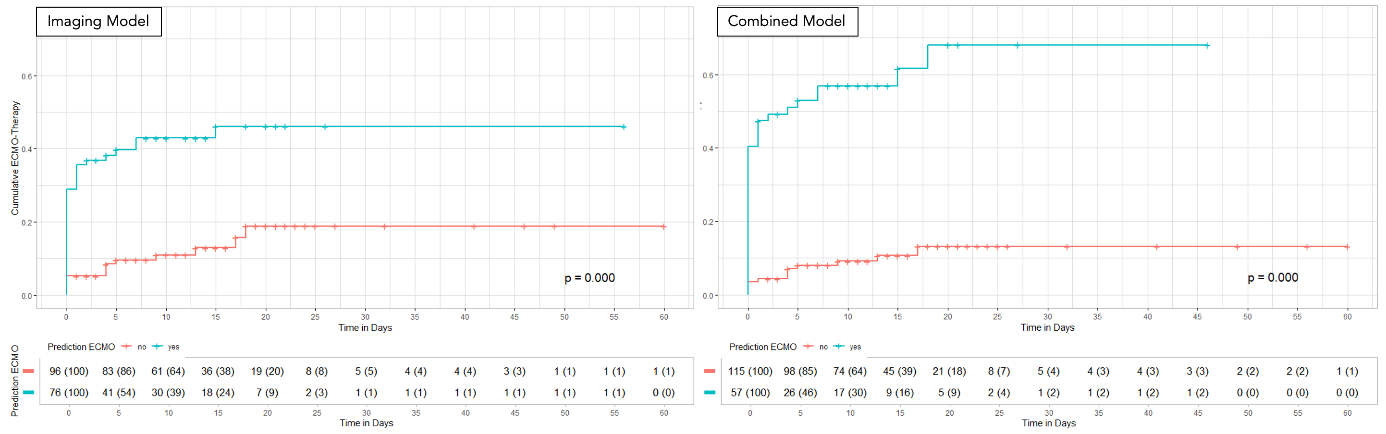
Figure S1: Kaplan–Meier curves for the cumulative incidence of vv-ECMO therapy in the training cohort, stratified by ECMO prediction.*
The figures illustrate the estimated cumulative probability of receiving vv-ECMO therapy over 60 days following ICU admission, stratified by ECMO prediction from the Imaging and Combined model, respectively. Patients predicted to require vv-ECMO are shown in turquoise; those predicted not to require ECMO are shown in red. The crosses indicate censored observations (discharge, transfer, or death). A statistically significant difference between the groups was observed (log-rank test, p < 0.001). The risk tables below show the number of patients at risk at each time point, with the percentage at baseline in parentheses.

**Supplement End**
